# Predictors of Exclusive Breastfeeding Amongst Mothers Living With HIV in Sub‐Saharan Africa: A Scoping Review

**DOI:** 10.1002/hsr2.73170

**Published:** 2026-09-09

**Authors:** Lydia Ives, Xinyi Ji, Mojisola Deborah Kupolati, Angel Macia Khoza, Phumudzo Tshiambara

**Affiliations:** ^1^ Well Being Africa Pretoria South Africa; ^2^ Department of Human Nutrition, Faculty of Health Sciences University of Pretoria Pretoria South Africa

**Keywords:** antiviral agents, breastfeeding, HIV, infant, nutrition, postnatal care, Sub‐Saharan Africa

## Abstract

**Background and Aim:**

Exclusive breastfeeding (EBF) reduces infant mortality and HIV vertical transmission (VT). The 2016 World Health Organisation (WHO) guidelines recommend 6 months of EBF for mothers living with HIV (MLWH) on antiretroviral therapy, yet uptake in sub‐Saharan Africa (SSA) remains low, and evidence on predictors is fragmented. This scoping review synthesised quantitative evidence on predictors of EBF among MLWH in SSA since the 2016 WHO guidelines, identifying geographic gaps and methodological trends to guide research and policy.

**Methods:**

Following Joanna Briggs Institute methodology and PRISMA‐ScR reporting, five databases, PubMed, EMBASE, Web of Science, Scopus, and Global Health, were searched for studies conducted from 2016 onwards that quantitatively assessed predictors of EBF among MLWH in SSA. Included studies were descriptively synthesised and mapped by geography, frequency, association, and analysis type. Quality appraisal used the Mixed Methods Appraisal Tool 2018.

**Results:**

19 studies met inclusion criteria. Positive predictors included healthcare engagement, knowledge, favourable attitudes, and HIV‐status disclosure. Negative/mixed predictors included employment, education, and pregnancy complications. Studies were geographically skewed (42% from Ethiopia), largely cross‐sectional (74%), and facility‐based. Psychosocial, socioeconomic, and biological/clinical barriers remain underexplored.

**Conclusions:**

Evidence on EBF predictors among MLWH in SSA remains limited, geographically uneven, and methodologically restricted. Future research should adopt longitudinal, intervention‐focused, and contextually diverse designs to inform scalable, culturally responsive strategies for improving EBF and preventing HIV VT.

## 1 Introduction

Human Immunodeficiency Virus (HIV) is a retrovirus that compromises immune function by targeting CD4 T cells, increasing susceptibility to infections and malignancies. Transmission occurs through bodily fluids, including vertical transmission (VT) of HIV during pregnancy, childbirth, and breastfeeding. Without antiretroviral therapy (ART), HIV can progress to acquired immunodeficiency syndrome (AIDS), a condition with high mortality [1].

Globally, in 2024, 40.8 million people were living with HIV; 31.6 million people received ART. 1.3 million people became newly infected with HIV, and there were 630,000 AIDS‐related deaths. In Eastern and Southern Africa alone, 21.1 million people are infected, with women and girls accounting for 63% of all cases [2]. Among pregnant and breastfeeding women in SSA, HIV incidence is estimated at 2.1 per 100 person‐years [3].

Exclusive breastfeeding (EBF) is defined by the World Health Organization (WHO) as feeding infants only breast milk for 6 months, except for medication/oral rehydration [4]. It is vital for infant immunity and development, as it reduces diarrhoea, respiratory infections, and malnutrition, the major causes of infant mortality in SSA [5, 6, 7]). Earlier WHO guidelines (2001) discouraged breastfeeding for mothers living with HIV (MLWH), but research later demonstrated that early mixed feeding raised HIV transmission risk and mortality [8, 9]). When MLWH begin ART before 30 weeks of gestation, and infants receive prophylaxis, the risk of HIV transmission decreases from approximately 3.5% to 1.9%–0.5% [10, 11]. In response, WHO updated its guidance in 2010 and 2016 to recommend that MLWH in low‐resource settings should practice EBF for 6 months in adherence to receiving ART, given the benefits on all‐cause mortality. The 2016 revision aimed to inform local programme implementation, health worker training, and counselling support [10]. The WHO also introduced Option B + , which recommends lifelong ART for all pregnant and breastfeeding MLWH [12].

Despite advances, EBF rates remain suboptimal. While the global target is 60% by 2030, SSA's rate is just 35.6% among MLWH, with variation by region and socioeconomic status [13, 14, 15]. Known barriers include HIV‐related stigma, outdated or contradictory guidance, cultural preferences for mixed feeding, family and partner influence, healthcare worker attitudes, inadequate knowledge, and fear of transmission [5, 16, 17, 18, 19]. Other constraints, including work demands, maternal illness, mental health, and food insecurity, are also reported [20]. There are gaps in the implementation of national policies. In South Africa, the 2010 Tshwane Declaration aligned with the WHO guidelines by discontinuing free infant formula for MLWH; however, the prior availability of formula influenced maternal behaviour, and EBF rates did not rise significantly [21, 22]. Many national policies across SSA vary widely, and their influence on maternal behaviour remains underexplored [23, 24].

Significant gaps persist in understanding EBF predictors among MLWH in SSA. Methodological inconsistencies further limit understanding. Studies often rely on cross‐sectional, self‐reported data, which may be subject to bias and cannot track sustained EBF behaviour over time. Much of the literature predates the WHO's 2010 and 2016 updates, leaving uncertainty about the translation of policy into practice. Key influences, including roles of fathers, extended family, and community support structures, are frequently overlooked [25]. Adolescent mothers, rural residents, and women with disabilities or low socioeconomic status are under‐represented in research [26].

Several studies isolate single predictors or lack examination of how social, economic, and structural factors interact [27]. Longitudinal studies are scarce, limiting insights into how practices evolve postnatally [28]. To date, no consensus exists on SSA data for EBF predictors until 6 months. Given the fragmented and diverse nature of the evidence base, a scoping review is the most appropriate design. Unlike systematic reviews, scoping reviews can accommodate varied study designs, map key concepts, and explore the extent and nature of existing research. This approach is suited to identifying evidence gaps, summarising emerging themes, and informing future research directions.

This scoping review synthesises quantitative evidence on the predictors among MLWH in SSA in the context of WHO's 2016 guidelines. It aims to identify which factors most consistently influence EBF, highlight understudied populations and geographical gaps, and summarise quantitative methodological trends. The findings will support researchers by guiding the design of consistent data collection tools for longitudinal research, enabling comparability across future systematic reviews and meta‐analyses. For policymakers and implementers, the review will clarify which predictor variables interventions should target, facilitating the development of culturally appropriate programmes that improve breastfeeding outcomes and prevent VT of HIV across diverse SSA contexts.

## 2 Key Messages

- The EBF reduces HIV transmission and supports infant survival, yet uptake among MLWH in SSA is low.
- Strengthening health service engagement, infant feeding counselling and education, and supporting HIV‐status disclosure can improve outcomes.
- Urgent, scalable interventions are required to inform policy, enhance services, and support breastfeeding for MLWH.

## 3 Methods

Scoping reviews aim to map existing literature in areas not yet systematically synthesised, present an overview of the topic, clarify key concepts, and identify research gaps [29]. This review followed the Joanna Briggs Institute (JBI) methodological guidance for the conduct of scoping reviews and was reported in line with the PRISMA‐ScR checklist for scoping reviews [Supporting Information I] [30, 31], and the protocol was registered on Open Science Framework in May 2025 (https://doi.org/10.17605/OSF.IO/UT325).

### 3.1 Eligibility Criteria

Eligibility was guided by the Population‐Concept‐Context (PCC) framework in accordance with the review's objectives [Table 1], with full criteria detailed in the protocol and applied at full‐text screening [Supporting Information II].

**Table 1:** Population, concept, and context for determining the scope of the research question.

Population	Mothers living with HIV
Concept	Predictors of the initiation/continuation of exclusive breastfeeding
Context	Sub‐Saharan Africa

The population included MLWH of any age who have given birth and had the opportunity to practice EBF. Only studies conducted in SSA, as defined by the World Bank, were included [Appendix I] [32]. Studies could focus on a specific country, region, or wider geographical area, utilizing data from any community or healthcare setting. A minor protocol modification indicated that primary data collection must have taken place from 2016 onwards to align with the updated WHO guidelines.

Eligible study designs included quantitative analytical methods (cross‐sectional, cohort, and case‐control) reporting measurable predictors and outcomes related to EBF. Qualitative studies were excluded to maintain focus on quantifiable variables and allow consistent data extraction of statistical measures of associations between predictors and EBF. Only peer‐reviewed articles with online full‐text availability and published in English were included.

### 3.2 Search Strategy

A three‐stage search strategy was developed collaboratively by the review team, who were nutrition and public health experts, to ensure comprehensive coverage of key concepts and minimise omissions.

A preliminary search in PubMed and EMBASE, which informed the final strategy [Supporting Information III] was conducted, followed by full search onPubMed, EMBASE, Web of Science, Scopus, and Global Health databases from April to May 2025 with afull list of key terms, full search strategies, and results is available in Supporting Information IV.

### 3.3 Article Screening

All citations were imported into EndNote 21 [EndNote V.21, Clarivate, Philadelphia, PA, USA, London, UK], and duplicate were removed. Citations were screened in Rayyan [Rayyan LLC, Dallas, Texas, TX, USA] based on title and abstract against the predefined PCC eligibility criteria and recording exclusion reasons by the primary reviewer. Records without abstracts advanced automatically to full‐text screening to avoid premature exclusion. Full texts were reviewed in Microsoft Excel using an 11‐item eligibility checklist, with inclusion/exclusion reasons recorded [Supporting Information V].

**Figure 1:**
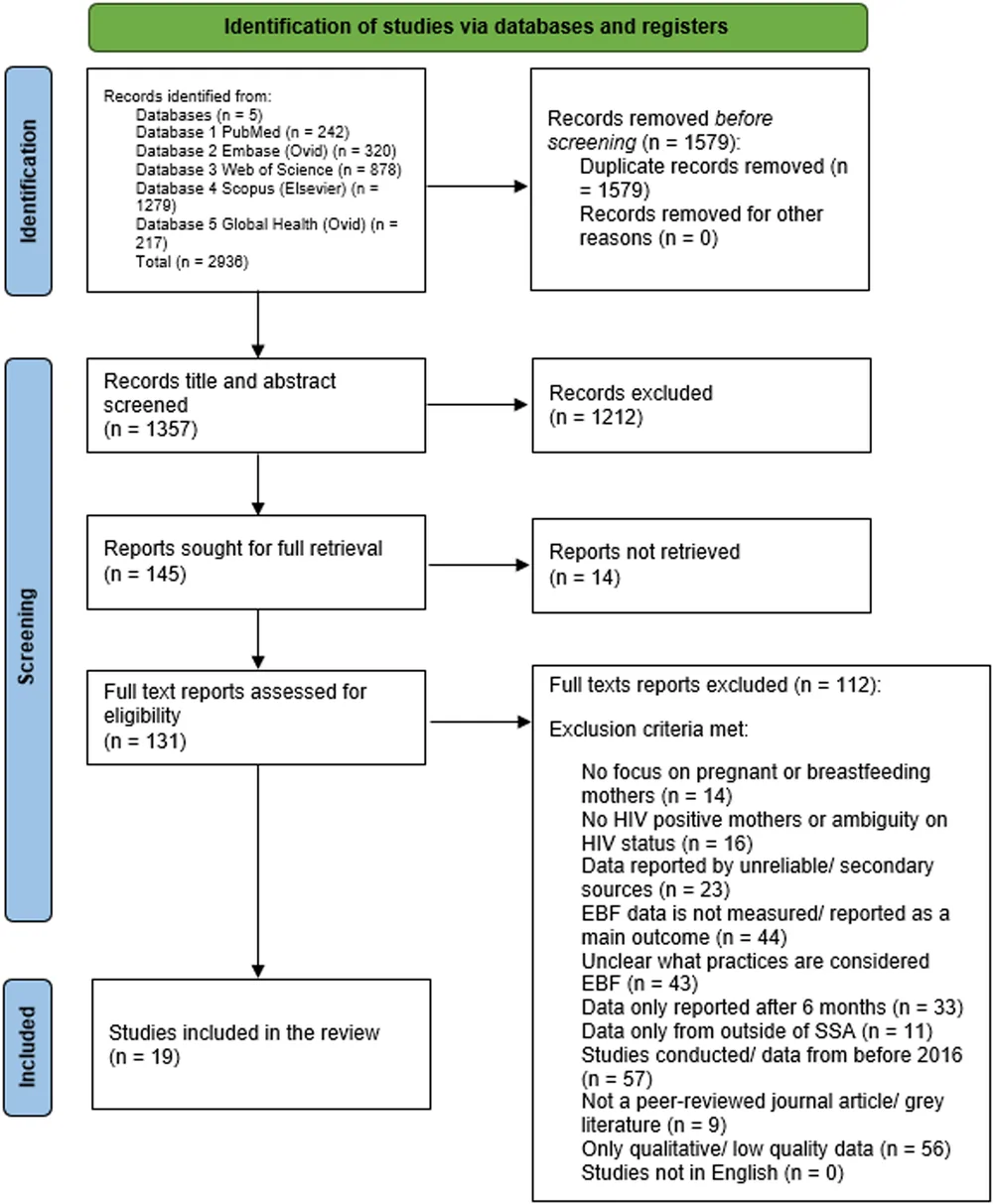
Predictors of exclusive breastfeeding amongst mothers living with HIV in sub‐Saharan Africa: a scoping review. Figure 1: PRISMA diagram illustrating the literature search, screening, and article selection.

The full study selection process is summarised in a PRISMA‐ScR flow diagram generated in Ryann [Figure 1] [33, 34].

### 3.4 Data Extraction

Data extraction was performed by the primary reviewer using a customized JBI template tool. Key information recorded, including authors, publication years, aims, and main findings. A second reviewer independently verified the data, with discrepancies discussed with a third reviewer. Study designs were assessed similarly, and disagreements were resolved through further discussion [Supporting Information VI]. The extracted data were organized for descriptive and narrative synthesis.

Data extraction involved a primary reviewer utilizing a customized JBI template tool. Key information such as authors, publication years, aims, and findings was recorded.

### 3.5 Data Synthesis

A descriptive and narrative synthesis was undertaken to address the review objectives while accommodating the heterogeneity of study designs, predictor variables, and outcome measures typical of scoping reviews. Study characteristics (country, year, and methodology) and key results were summarised, with frequency counts used to describe study designs. Geographic distribution was illustrated using a heat map. The 2018 Mixed Methods Appraisal Tool (MMAT) was used to assess study quality [35] with study quality was scored as low (≤ 50%), average (51%–75%), or high (76%–100%.).

### 3.6 Ethical Considerations

Ethical approval and informed consent were not required. This scoping review relied on a synthesis of the existing literature from publicly available, peer‐reviewed journal articles.

## 4 Results

### 4.1 Screening Results

From 2,963 records identified across five databases (April–July 2025), 1,375 titles/abstracts remained after deduplication and were screened in Rayyan. A total of 1,212 were excluded due to incorrect population (*n* = 715), concept (*n* = 1,134), context (*n* = 102), or pre‐2016 data (*n* = 180), with some meeting multiple criteria. No articles were excluded for language. Abstracts were unavailable for 32 records, which proceeded to full‐text screening. Of 145 full texts sought, 132 were retrieved; 13 without accessible digital copies were excluded. All studies considered to have potential eligibility by the primary reviewer (*n* = 28) were independently assessed by a second reviewer. Following full‐text screening, nine studies were excluded due to qualitative study design (*n* = 6), data collected only before 2016 (*n* = 2), or lack of full‐text access (*n* = 1), and both reviewers agreed on the final inclusion of 19 articles using an 11‐item checklist [Supporting Information V].

### 4.2 Article Characteristics

Table 2 summarises each study's population, setting, outcome, and characteristics (author, year, title, aims/outcomes, country, setting, study design, and sample size). Quantitative findings are also presented narratively.

**Table 2:** Characteristics and findings of studies included in the scoping review.

Author and year	Title	Aim/outcome	Country	Setting	Study design	Sample size	Main findings
Napyo et al., 2020	Exclusive breastfeeding among HIV‐exposed infants from birth to 14 weeks of life in Lira, Northern Uganda: A prospective cohort study.	Assess the risk factors for non‐EBF in the first 14 weeks postpartum among MLWH	Lira district ‐ Northern Uganda	Prevention of VT of HIV clinics, Lira Regional Referral Hospital	Quantitative prospective cohort study	*N* = 466	Most initiated EBF at birth (87.3%), and a few practised prelacteal feeding; proportions declined by 6 and 14 weeks (77.5% & 57.1%). In multivariable analysis, continued EBF at 14 weeks was positively associated with lower socioeconomic status, health worker delivery, and ART adherence.
Mekonnen et al., 2022	Infant feeding practices and their associated factors among HIV positive mothers attending public health institutions at Gondar Town, Northwest Ethiopia, 2019: An institution‐based based cross‐sectional study	Assess infant feeding practices and associated factors among MLWH	Gondar Town ‐ Northwest Ethiopia	Public health institutions	Quantitative cross‐sectional study	*N* = 355	Most practised EBF (87.4%), early initiation (79.4%), and complementary feeding after 6 months (94%). In multivariable analysis, recommended breastfeeding practices (EBF/ERF) were positively associated with younger age, rural residence, social support, no HIV status disclosure, absence of pregnancy complications, lack of ANC follow‐up, and home delivery.
Mebratu et al., 2020	Exclusive Breast‐Feeding Practice and Associated Factors among HIV‐Positive Mothers in Governmental Health Facilities, Southern Ethiopia	Assess EBF prevalence and associated factors among MLWH attending prevention of VT of HIV clinics	Southern Ethiopia	Government prevention of VT of HIV clinics	Quantitative cross‐sectional study	*N* = 209	Most had good knowledge of prevention of VT of HIV and infant feeding and expressed favourable attitudes (80%). Early initiation and EBF are common (81.6%), though some introduced solids before six months (11.6%) or stopped before 12 months (8.6%) due to transmission fears and inappropriate advice. In multivariable analysis, EBF was positively associated with ≥ 4 ANC visits, HIV status disclosure, good knowledge, and positive attitudes.
Dagnew et al., 2019	Factors Associated with Compliance with World Health Organization‐Recommended Infant‐Feeding Practices by Mothers with HIV Infection in Northwest Ethiopia	Assess the factors associated with noncompliance to WHO† breastfeeding recommendations among MLWH‡, focusing on infant feeding practices and predictors	Bahir Dar City ‐ Northwest Ethiopia	Government hospitals or health centres providing prevention of VT§ of HIV clinics and ART services	Quantitative cross‐sectional study	*N* = 213	ERF†† prevalence was 11.2% higher than EBF‡‡ prevalence; early initiation (< 1 h) was widely practised. 69% initiated breastfeeding within 1 h of birth. EBF/ERF was positively associated with higher maternal education, ANC§§ and PNC¶¶ attendance, and HIV status disclosure to a spouse at the multivariate level.
Faustine & Moshi, 2022	Exclusive breastfeeding practice among HIV infected mothers in the southern highlands of Tanzania: assessing the prevalence and factors associated with the practice, an analytical cross‐sectional survey	Assess EBF prevalence and associated factors among MLWH	Iringa and Njombe regions – Southern Highlands of Tanzania	Health institutions' prevention of the VT of HIV programmes	Quantitative cross‐sectional study	*N* = 372	Just over half practised EBF (58.1%); adequate knowledge and positive attitudes were moderate. In multivariable analysis, EBF was positively associated with adequate knowledge, ANC attendance, income ≥ 1 USD/day, positive attitudes, and absence of breastfeeding problems.
Iliyasu et al., 2019	Determinants of Infant Feeding Practices Among HIV‐Infected Mothers in Urban Kano, Nigeria	Assess the knowledge and predictors of appropriate infant feeding practices among MLWH	Urban Kano ‐ Northern Nigeria	S. S. Wali HIV Centre at Aminu Kano Teaching Hospital ‐ Tertiary health facilities	Quantitative cross‐sectional study	*N* = 203	Awareness of 6‐month EBF and ART recommendations was moderate, and HIV transmission risks were low; over two third participants practised EBF for six months, others mixed‐fed or switched to ERF. In multivariate analysis, EBF was positively associated with counselling and hospital delivery.
Zewdu et al., 2023	Unsafe Infant Feeding Practices and Associated Factors Among HIV‐Positive Mothers Attending PMTCT in Ethiopia: A Cross‐Sectional Study	Assess unsafe infant feeding practices and associated factors among MLWH attending prevention of VT of HIV clinics	Afar regional state ‐ Ethiopia	Prevention of VT of HIV clinics at governmental hospitals	Quantitative cross‐sectional study	*N* = 423	The majority practised EBF (63.8%), and some mixed‐fed (36.2%); no ERF was reported. In multivariable analysis non‐EBF was positively associated with no PNC follow‐up, no ART follow‐up, and non‐disclosure of HIV status.
Zunza et al., 2023	Evaluating interactive weekly mobile phone text messaging plus motivational interviewing for breastfeeding promotion among women living with HIV, giving normal birth at a primary healthcare facility in South Africa: A feasibility randomised controlled trial	Assess the feasibility of recruiting and retaining participants in a randomised trial, and gathered preliminary data on EBF rates following a mobile text messaging plus motivational interviewing intervention	Breede Valley ‐ South Africa	Worcester Midwife Obstetric Unit	Randomised controlled feasibility trial	*N* = 52	Higher EBF at week 24 in the intervention group (weekly text + motivational interviewing) vs control (77.8% vs 55.6%), but the difference was not significant.
Mukhula et al., 2025	Comparison of infant feeding practices by maternal HIV status, and associated factors, in a rural district, South Africa, 2019	Compare infant feeding practices by maternal HIV status, assessing associated factors in MLWH and HIV‐negative mothers	Ehlanzeni district, Mpumalangaprovince – North‐East South Africa	Community healthcare centres	Secondary data analysis from a cross‐sectional study	*N* = 771 (478 HIV‐positive mothers, 143HIV‐exposed infants aged 0–6 months)	The highest prevalence of EBF was found in HIV‐exposed infants aged 0–3 months (41.0%), with lower prevalence in HIV‐exposed infants aged 3–6 months (16.7%). In bivariable analysis, in HIV‐exposed infants aged 0–6 months, attending ≥ 5 ANC visits was positively associated with a lower likelihood of EBF.
Muhammed & Seid, 2019	Determinants of none‐exclusive breast‐feeding practice among HIV positive women at selected Health Institutions in Ethiopia: case‐control study	Identified socioeconomic and health determinants of non‐EBF among MLWH	Dessie town, Amhara region – Northern Ethiopia	Public health facilities	Unmatched case‐control study	*N* = 249	In multivariable analysis, non‐EBF was positively associated with employment, secondary education, and home delivery.
Goon et al., 2021	Sociodemographic and lifestyle correlates of exclusive breastfeeding practices among mothers on antiretroviral therapy in the Eastern Cape, South Africa	Assess EBF prevalence and associated factors among MLWH on ART	Eastern Cape ‐ South Africa	Maternity facilities at Frere, Cecilia Makiwane, and Bisho hospitals	Quantitative cross‐sectional study	*N* = 469	About one‐third practised EBF for six months (32.0%). In multivariable analysis EBF was positively associated with unemployment, education ≤ grade 12, and no alcohol consumption.
Etowa et al., 2021	Determinants of infant feeding practices among Black mothers living with HIV: A multinomial logistic regression analysis	Assess the determinants of infant feeding practices among Black MLWH on ART in two North American cities and one African city, examining the influence of knowledge, attitudes, social support, and health education	Port Harcourt ‐ NigeriaOttawa, CanadaMiami, Florida, US	Community resource centres, public health facilities, and AIDS service organisations	Quantitative cross‐sectional study	*N* = 690 Port Harcourt (*n* = 400) Ottawa (*n* = 89) Miami (*n* = 201)	Multinomial models account for national guidelines (EBF in Nigeria vs ERF in Canada/US). Mothers generally held positive attitudes. In Nigeria, 66.7% practised EBF; others practised ERF (18.1%) or mixed feeding (15.2%). Most were on ART, attended ANC, and had received infant feeding advice. Partner support for EBF was common; the wider family had a lesser influence. In multivariable analysis, receiving care during pregnancy, receiving care from nurses or midwives, a more positive attitude towards breastfeeding, higher functional social support, and lower stress levels were positively associated with EBF over mixed feeding.
Genetu et al., 2016	Breastfeeding counselling and support are associated with continuous exclusive breastfeeding from 1 week to 6 months of age among HIV exposed infants in the north Gondar zone, Ethiopia: A cross‐sectional study	Assess EBF rates from 1 week to 6 months among MLWH and associated factors.	North Gondar Zone ‐ Northwest Ethiopia	HIV clinics at government hospitals	Quantitative cross‐sectional study	*N* = 367	Most practised EBF from 1 week to 6 months (86.4%), early initiation was common (72.8%), and prelacteal feeding was rare (19.1%). In multivariable analysis, EBF was positively associated with prenatal counselling, breastfeeding support, and absence of obstetric complications.
Bultum et al., 2022	Factors influencing exclusive breastfeeding among children born to HIV positive mothers attending public health facilities in western Ethiopia: Cross‐sectional study	Assess the factors influencing EBF among MLWH	West, East and Kellem Wollega districts – Ethiopia	Prevention of VT in HIV and ART clinics at public health facilities	Quantitative cross‐sectional study	*N* = 219	Just over half practised EBF (56.0%), with most initiating breastfeeding within 1 h of delivery (61.8%). Most attended PNC (95.9%) and had received EBF advice (22.5%). Most knew HIV could be transmitted via breast milk (82.0%); few recognised risks during pregnancy, delivery, or breastfeeding; and a quarter were unaware that EBF protects against infant diarrhoea (28.4%). In multivariable analysis, EBF was positively associated with receiving EBF advice, disclosing HIV status, and believing HIV can be transmitted during delivery.
Zunza et al., 2025	A randomized controlled trial on the effects of mobile phone text messaging in combination with motivational interviewing versus standard infant feeding counselling on breastfeeding and child health outcomes, among women living with HIV	Evaluate whether text messaging plus motivational interviewing prolongs EBF and improves child health outcomes up to 24 weeks.	Khayelitsha, Cape Town – South Africa	Khayelitsha District Hospital and follow‐up at Masiphuhlisane Research Centre	Randomized control trial	*N* = 276	Fewer than 10% of mothers in either the intervention (text messages + motivational interviews) or control groups practised EBF at 24 weeks; median cessation was 16 weeks. No significant intervention effects. Stopping reasons were described as work, job search, insufficient milk, and infant refusal.
Mutawulira et al., 2022	Exploring infant feeding practices and associated factors among HIV‐positive mothers attending the early infant diagnosis clinic in Northern Uganda	Assess infant feeding practices and determinants among MLWH with infants aged 0–12 months.	Gulu District – Northern Uganda	Awach Health Centre	Mixed methods cross‐sectional study	*N* = 108	Most practised EBF for six months (77%), and others practised mixed feeding (23%); knowledge of HIV transmission was high (96.3%), but detailed understanding was limited. In multivariable analysis, EBF was positively associated with younger child age, higher income, fewer early infant diagnosis clinic visits, and facility‐based delivery.
Gejo et al., 2019	Exclusive breastfeeding and associated factors among HIV positive mothers in Northern Ethiopia	Assess the magnitude of EBF and associated factors among MLWH, focusing on knowledge, attitudes, counselling, and HIV status disclosure	Tigray region – Northern Ethiopia	ART and PMTC at public hospitals	Quantitative cross‐sectional study	*N* = 304	Most practised EBF for six months (88.8%), and others practised ERF (4.6%) or mixed feeding (6.6%). Most demonstrated strong knowledge of HIV transmission (79.9%). Favourable attitudes and high rates of counselling and HIV status disclosure were common; over 90% initiated breastfeeding within 1 h of birth. In multivariable analysis, EBF was positively associated with counselling, good knowledge, and positive attitudes. Fear of HIV transmission motivated EFF.
Philemon et al., 2022	Adherence to Optimal Breastfeeding Practices Among HIV‐Positive Mothers in Kilimanjaro, Tanzania	Assess adherence to optimal breastfeeding practices among MLWH enrolled in prevention of VT of HIV programmes, including early initiation, EBF to six months, and continued breastfeeding to one year, and identify associated factors.	Kilimanjaro – Tanzania	Prevention of VT in HIV clinics	Quantitative cross‐sectional study	*N* = 524	Three‐quarters initiated within 1 h (73.1%); prelacteal feeding was rare (2.3%). In multivariable analysis, 6‐month EBF was positively associated with ≥ 2 pregnancies while HIV positive, ≤ 5 years of HIV duration, < 4 ANC attendances, and hospital‐level ANC attendance; early initiation was positively associated with spontaneous vaginal delivery, normal birth weight, ≥ 2 pregnancies while HIV positive, and no disclosed HIV serological status to partner; continued breastfeeding to 12 months was positively associated with ART counselling, ≥ 2 pregnancies while HIV positive, shorter years on ART, no breastfeeding support after delivery, and successful EBF.
Ejara et al., 2018	Inappropriate infant feeding practices of HIV‐positive mothers attending PMTCT services in Oromia regional state, Ethiopia: A cross‐sectional study	Assess the prevalence and predictors of inappropriate infant feeding among MLWH, focusing on knowledge, attitudes, counselling, and ANC/PNC attendance.	Adama and Bushoftu towns, Oromia, Ethiopia	Prevention of VT of HIV services at public health institutions	Quantitative cross‐sectional study	*N* = 283	Most practised EBF for six months (83.5%), 6.7% EFF, 8.3% mixed feeding; most had high knowledge (93.0%); majority initiated within 1 h (69.7%) In multivariable analysis, EBF was positively associated with favourable attitudes, ANC/PNC attendance, counselling, HIV status disclosure, and absence of breastfeeding problems or infant mouth ulcers.

*Note:* † WHO = World Health Organization, ‡ MLWH = mothers living with HIV, § VT = prevention of mother‐to‐child transmission, ¶ ART = antiretroviral therapy, †† ERF = exclusive replacement feeding, ‡‡ EBF = exclusive breastfeeding, §§ ANC = antenatal care, ¶¶ PNC = postnatal care.

Data were predominantly collected in health facilities via semi‐structured questionnaires, with sample sizes ranging from 52 to 690, mainly among postpartum MLWH attending health services. Almost all studies reported descriptive statistics; one used only bivariate analysis; others applied bivariate models to inform multivariate logistic regressions.

### 4.3 Study Geographical Distribution

**Figure 2:**
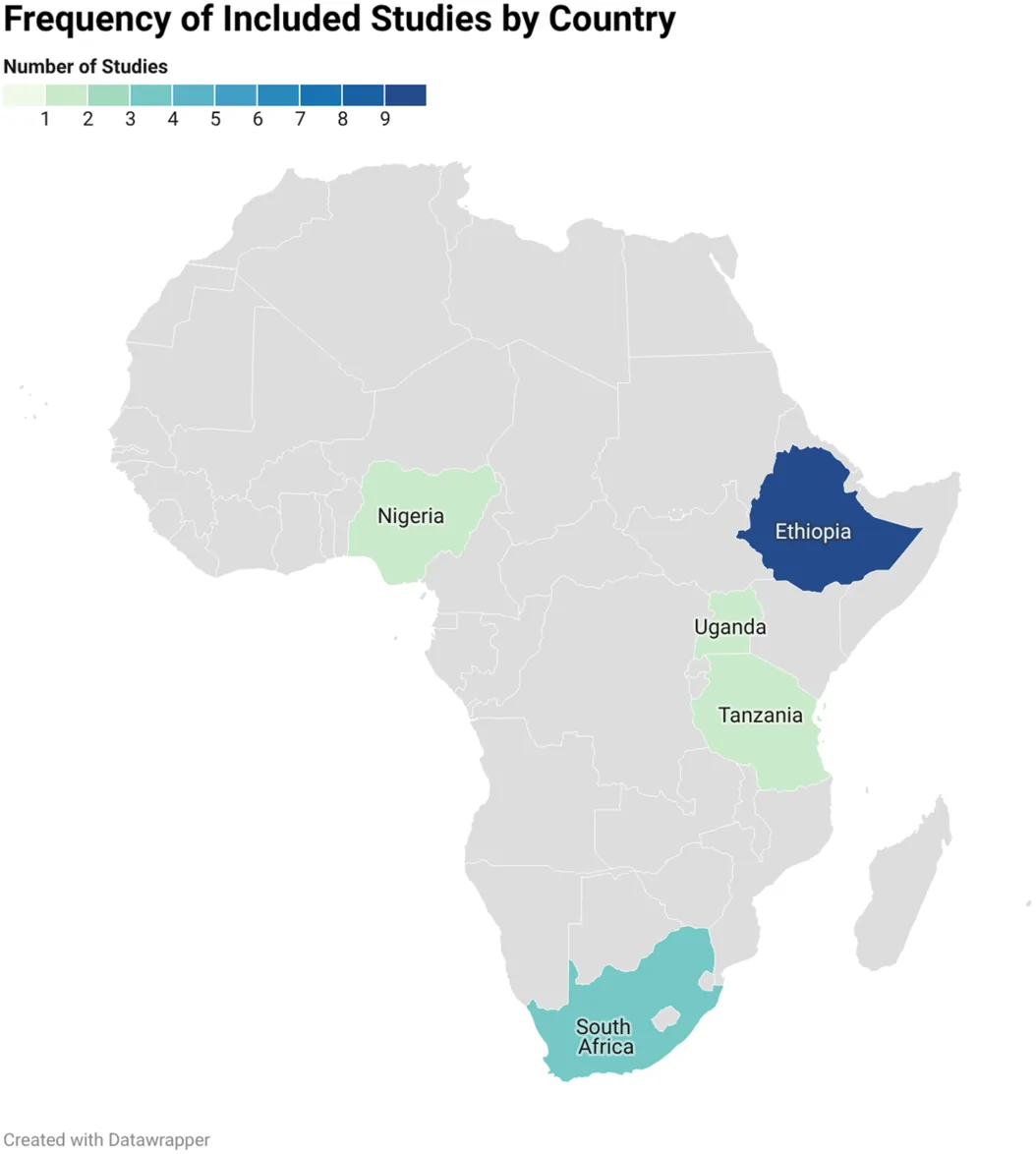
Heat map of study frequency by country.

Studies were conducted in five countries, with Ethiopia contributing nearly half (*n* = 9). Others were from South Africa (*n* = 4), Nigeria (*n* = 2), Tanzania (*n* = 2), and Uganda (*n* = 2). Most were in Eastern Africa (*n* = 13), then Southern (*n* = 4) and Western Africa (*n* = 2); none were from Central Africa (Figure 2).

#### 4.3.1 Quality of Evidence

Methodological quality was generally high (17 high, 1 average, 1 low) [Supplementary Information VII]. Following the MMAT tool, 19, included studies were published between 2016 and 2025, with a notable decline from 2023 onwards. Fourteen studies used a cross‐sectional design, one conducted secondary data analysis of cross‐sectional data, and the remaining four used longitudinal designs: 1 feasibility RCT, 1 RCT, 1 case‐control study, and 1 prospective cohort study.

#### 4.3.2 Main Findings

A total of 24 unique significant predictors of EBF were examined across 19 studies and summarised in Table 2. A breakdown of predictors was also included in Supporting Information VIII.

Most studies compared EBF with non‐EBF, while two studies grouped EBF and exclusive replacement feeding (ERF) as recommended feeding practices [36, 37], and three studies compared EBF with mixed feeding [38, 39, 40].

The most frequently studied predictors were related to healthcare access, followed by maternal knowledge, psychosocial, socioeconomic, and clinical/biological factors. Antenatal care (ANC) attendance was most frequently assessed (*n* = 8; 75% positive). Other consistent positive predictors were maternal knowledge (*n* = 5), HIV‐status disclosure (*n* = 6), infant feeding counselling (*n* = 5), and favourable attitudes. (*n* = 4). Negative predictors, including breast problems, maternal employment, pregnancy complications, alcohol use, frequent early infant diagnosis visits, child age, mouth ulcers, and maternal stress, were infrequently studied. Higher education (*n* = 3; 33% positive), hospital delivery (*n* = 4; 75% positive), and high income (*n* = 3; 66% positive) showed mixed associations. Evidence for negative and mixed predictors remains limited.

#### 4.3.3 Healthcare Engagement

Healthcare engagement, particularly ANC attendance and infant feeding counselling, was the most common predictor in both cross‐sectional and longitudinal studies. ANC attendance was significantly positively associated with EBF or EBF/ERF in five studies [37, 38, 40, 41, 42], while multiple ANC attendances were significantly negatively associated with EBF in 3 studies [36, 43, 44].

Postnatal care (PNC) attendance was positively associated with EBF but was less frequently studied than ANC [37, 40, 45]. Delivery attended by a health worker was also a positive predictor [46]. Conversely, frequent early infant diagnosis clinic visits were linked to lower EBF rates [39]. Findings on hospital delivery were mixed, with most studies reporting positive associations with EBF [39, 47, 48], and one reporting negative associations with EBF/ERF despite high overall EBF rates [36].

##### 4.3.3.1 Knowledge and Attitudes

Knowledge and attitudes toward EBF were assessed in four studies and showed strong positive associations with EBF outcomes. However, definitions of “adequate knowledge” varied, using either predefined cut‐offs [41, 49] or mean‐based thresholds [42]. Mean‐based thresholds were sample‐dependent, while predefined cut‐offs, though clearer, lacked consistency and standardisation. Adequate knowledge varied substantially across studies, with Mebratu et al [41] identifying knowledge of infant feeding as the only factor associated with EBF. Awareness of HIV transmission risks during pregnancy, delivery, and breastfeeding ranged from 23% in Bultum et al [50] to 80% in Gejo et al [49], highlighting marked differences across settings. Five studies found favourable attitudes positively associated with EBF, with rates ranging from 52% to 84% [38, 41, 42, 45, 49]. In three of the five studies, both knowledge and attitudes were significant predictors [41, 42, 49]. Overall, despite methodological variation, knowledge and attitudes were among the most consistently studied and strongly positive predictors of EBF.

##### 4.3.3.2 Psychosocial Predictors

HIV‐status disclosure was a significant positive predictor of EBF in six studies [36, 37, 40, 41, 50], with reported rates of 64%–95%. Definitions ranged from spouse‐only to community disclosure. All disclosure studies were conducted in Ethiopia. Other psychosocial variables were less consistently studied. Social support appeared in two studies, with one reporting 14% global support [36] and another reporting 59% on a validated functional support scale [37]; both were positively associated with EBF. Alcohol abstinence [5] was positively associated with EBF in one study. Perceived stress was negatively associated with EBF over mixed feeding in one study [38].

##### 4.3.3.3 Socioeconomic Predictors

Socioeconomic predictors exhibited inconsistent associations. Employment was a significant negative predictor of EBF in Ethiopia (39% employed) and in South Africa (63% employed) [5, 48]. In the same studies, higher education also predicted lower EBF. Conversely, Dagnew et al [37] found high school education or above positively associated with EBF in Ethiopia, despite finding lower overall levels of education.

Income showed conflicting directionality. It was positively associated in studies from Tanzania and Uganda [39, 42] but negatively associated in a Ugandan cohort using a broader asset‐based index [46]. In addition, a rural Ethiopian sample found that rural residence was positively associated with EBF/ERF [36].

### 4.4 Biological and Clinical Predictors

Breast issues and infant mouth ulcers were negatively associated with EBF in two studies [42, 45]. Pregnancy or obstetric complications were also negative predictors: 7% of mothers with unspecified issues [36], and 47% with obstetric complications, both linked to reduced EBF and higher infant HIV infection [51] in Ethiopia.

Positive predictors of EBF included adherence to ART and engagement in its services, supported by two studies showing a correlation between these factors and higher EBF rates [39, 40], aligning with WHO 2016 guidelines [10]

Parity and duration of HIV diagnosis demonstrated mixed associations within the same study. Philemon et al. (2022) [44] reported higher adherence among mothers diagnosed within the previous 5 years, as well as among those who had two or more children while living with HIV. Meanwhile, Mekonnen et al [36] reported the highest adherence among mothers aged 25–29, followed by 18–24.

#### 4.4.1 Clinical Interventions

We identified two clinical intervention studies: one RCT and one feasibility RCT. Both studies tested mobile text messaging plus motivational interviewing versus standard infant nutrition counselling in different cohorts of MLWH [8, 52]. Neither showed statistically significant effects on EBF.

Overall, studies identified a range of EBF predictors among MLWH, with positive predictors more frequently and consistently reported, while context‐specific and negative biopsychosocial factors remain underexplored.

## 5 Discussion

This scoping review examined predictors of EBF among MLWH in SSA in line with WHO's 2016 guidelines, aiming to identify consistently reported predictors and geographic/methodological gaps. To meet the aim, we restricted inclusion to quantitative studies reporting associations between predictors and EBF outcomes, excluding qualitative studies that do not provide comparable effect estimates. EBF protects infant health and reduces HIV vertical transmission, yet uptake among MLWH in SSA remains low, and fragmented evidence on its determinants hinders targeted interventions [4, 53]. Key trends emerged around health service engagement, maternal knowledge, and psychosocial factors, while fewer studies addressed biological, clinical, or negatively associated predictors.

### 5.1 Healthcare Engagement

Healthcare engagement, particularly ANC, PNC, and hospital births, was the most studied and consistently positive predictor of EBF, often acting as a proxy for access to education and provider trust [37, 41, 45]. Frequent attendance may reflect mothers' perceived value of care. However, some studies found negative associations with high ANC or early infant diagnosis visits, likely due to underlying maternal health needs, underscoring the need to distinguish engagement from care‐seeking for complications [39, 43]. Most studies used a four‐visit ANC benchmark despite the WHO's eight‐visit recommendation, highlighting limited uptake and interpretation [54]. In a South African cohort with high ART access, only 49% of HIV‐exposed infants were breastfeeding at 6 months versus 64% of unexposed peers, showing that service access alone does not ensure adherence [19]. Healthcare engagement may lead to early initiation of EBF, but does not necessarily ensure its continuation once it has begun [55].

Infant feeding counselling was among the most reliable positive predictors, though inconsistent or outdated advice and provider biases were noted [36, 38]. Counselling quality, rather than exposure, may better predict EBF outcomes [44, 47].

#### 5.1.1 Knowledge and Attitudes

Despite inconsistent measurement thresholds, maternal knowledge and favourable attitudes toward EBF were consistently significant predictors, supporting autonomy in decision‐making [38, 41, 42, 49]. Knowledge and attitudes often co‐occurred, aligning with health belief models emphasising perceived risk, benefit, and self‐efficacy in adherence [56]. Adherence likely improves when mothers understand the rationale for recommendations [38].

However, knowledge alone is insufficient without cultural acceptability. Cultural practices such as giving water in hot weather or honey for colic, often endorsed by relatives, can perpetuate mixed feeding [38]. Stigma may occur regardless of the feeding method [38]. From a health belief model perspective, peer influence could counter this by modelling EBF and highlighting mixed feeding risks [44]. Notably, stigma itself was not a significant independent predictor [57].

#### 5.1.2 Psychosocial Predictors

HIV‐status disclosure was the most consistently positive psychosocial predictor of EBF, potentially reducing psychological burden, enabling support, and avoiding concealment through alternative feeding [37, 41, 45]. Non‐disclosure to healthcare providers may restrict appropriate care [40]. However, studies rarely considered the disclosure recipient or relationship quality. Social support, also a positive predictor, may mediate disclosure. Other psychosocial factors, such as stress, alcohol use, and religion, were seldom assessed despite their likely relevance.

#### 5.1.3 Socioeconomic Predictors

Income, education, and employment were interlinked [46]. Education may increase EBF knowledge [37, 39] but also employment likelihood, reducing time with infants and healthcare access. Despite legislation, awareness of workplace rights is low, and expressing milk has limited acceptance [5].

Higher income may facilitate formula use, while poverty can force mixed feeding [45]. Studies reporting higher EBF among urban or rural mothers primarily included participants from those areas, limiting generalisability. In rural contexts with strong outreach, close ties with community health workers, and limited formula access may support EBF [36, 39].

#### 5.1.4 Clinical and Biological Predictors

Breast health issues and obstetric complications were negative predictors in isolated studies [42, 51], potentially delaying EBF initiation and undermining maternal confidence. Many are preventable with early intervention [45]. ART adherence was positively associated with EBF, reflecting reduced transmission fears and greater exposure to guidelines [40, 41]. In a South African cohort, breast milk composition at 6 months was similar between MLWH and those without HIV, suggesting that nutritional adequacy is unlikely to be a reason for discontinuing breastfeeding [58]. By this age, HIV‐exposed infants already showed poorer growth, underscoring the need to protect breastfeeding in this vulnerable group [19].

While some clinical and biological factors overlap with healthcare access, they warrant independent examination.

### 5.2 Heterogeneity of EBF Outcomes

The WHO defines EBF as the provision of only breastmilk to infants from birth until 6 months, barring medical advice [4, 13]. Key EBF indicators include early initiation of breastfeeding within an hour after birth and assessment of EBF status for infants aged 0–6 months based on their exclusive milk intake in the previous 24 h [59]. Differences in the timing and influencing factors suggest that predictors for early initiation may differ from those for maintaining EBF or current status. These predictors may reflect the MLWH's ability to sustain EBF even when they are less predictive of initial uptake [59]. These factors may show limited association with ever initiating EBF but substantial effects on maintaining EBF up to 6 months.

The EBF predictors in MLWH include education, healthcare engagement, social support, psychological well‐being, and socio‐cultural factors. Variations in study results highlight contextual and methodological differences. Future research should focus on psychosocial interventions, family involvement, culturally sensitive counselling, and longitudinal designs to improve breastfeeding outcomes and support global vertical transmission initiatives [60, 61].

### 5.3 Implications for Policy and Practice

ANC and PNC counselling should deliver standardised, evidence‐based messages aligned with WHO guidance, provided by trained staff [43]. Integrating feeding advice into ART services can support both virological suppression and infant nutrition. Given the influence of peers and family, community interventions should address norms, cultural acceptability, and stigma.

Workplace protections need to be enforced, along with education on expressing breastmilk and awareness of entitlements. Psychosocial support for disclosure, stress, and substance use should be integrated into services. Linking facility and community approaches is essential for reaching underserved mothers. Innovative, multidimensional strategies are needed to drive lasting behaviour change.

### 5.4 Strengths and Limitations

This scoping review comprehensively examined EBF predictors among MLWH in SSA since the 2016 WHO guidelines. It followed a comprehensive search strategy and clear eligibility criteria, effectively leveraging a range of keywords and medical subject headings. All inclusion and exclusion procedures were transparent, with a second reviewer involved in validating included texts. The exclusion of qualitative studies improved the consistency in data extraction and comparability of measurable predictors across the studies.

Limitations stem from the included studies: predominant use of observational studies that cannot perform causal inferences, reliance on non‐standardised self‐reports, varied EBF definitions, focus solely on MLWH without other informants, and likely social desirability and recall bias, especially post‐counselling. No risk of bias assessment or advanced synthesis was performed, consistent with scoping methodology, limiting interpretation of effect sizes and directionality. The studies' selection was primarily conducted by a single researcher, introducing potential selection or interpretive bias; however, a second reviewer and a third reviewer were involved in the final decisions. Also, English restriction and exclusion of grey literature might have ignored relevant data. Restricting inclusion to quantitative studies limited the contextual understanding of EBF.

### 5.5 Recommendations for Future Research

Future research should utilize longitudinal and interventional designs to better understand non‐EBF adherence and the various behavioural drivers involved. There is a need for standardized, validated tools for data collection, including the IYCF indicators, to enhance comparability in reviews [59]. Adaptation of WHO guidelines on feeding practices is essential, focusing on region‐specific tools to assess mothers' knowledge and attitudes regarding EBF and HIV prevention. Research should also increase geographic coverage, especially in Central Africa, and involve vulnerable groups to ensure equity. Qualitative studies uncover cultural beliefs against EBF, while the effects of socioeconomic factors, including food insecurity, should be investigated. Mixed‐methods approaches are recommended to integrate qualitative insights with quantitative data, informing tailored interventions. Additionally, more comprehensive evaluations of WHO breastfeeding guidelines are necessary, and trials to identify effective strategies in diverse contexts are urgently needed.

## 6 Conclusion

In conclusion, this scoping review highlights a limited and uneven body of evidence on EBF among marginalized MLWH in SSA, with no studies from Central Africa and few studies from high‐burden areas. Most research was cross‐sectional and facility‐based, focusing on positive predictors including healthcare engagement and maternal knowledge, while neglecting psychosocial and socioeconomic barriers. Interventional studies are scarce, indicating a need for geographically diverse, longitudinal research to develop effective, culturally sensitive strategies for enhancing maternal and child health, including in the context of HIV.

## Author Contributions

L.I. conceptualised and wrote the draft manuscript under the supervision of P.T. and M.D.K. P.T. contributed to the development of the search summary table. L.I. optimised the search strategy and performed the literature search. X.J. and A.M.K. contributed to the title and abstract screening, as well as the full‐text screening of the eligible articles. X.J., P.T., and M.D.K. critically reviewed and provided input to revise the manuscript. All authors have read and approved the final version of the manuscript. Dr Phumudzo Tshiambara had full access to all of the data in this study and takes complete responsibility for the integrity of the data and the accuracy of the data analysis.

## Funding

The authors have nothing to report.

## Conflicts of Interest

The authors declare no conflicts of interest.

## Transparency Statement

The lead author, Lydia Ives, affirms that this manuscript is an honest, accurate, and transparent account of the study being reported; that no important aspects of the study have been omitted; and that any discrepancies from the study as planned (and, if relevant, registered) have been explained.

## Supporting information


Supporting File 1

Supporting File 2

Supporting File 3

Supporting File 4

## Data Availability

Data sharing is not applicable to this article, as no datasets were generated or analysed during the current study.
