# Supplementary material for: Predictors of Exclusive Breastfeeding Amongst Mothers Living With HIV in Sub‐Saharan Africa: A Scoping Review
Source: Health Sci Rep. 2026 Sep 9;9(9):e73170. doi: 10.1002/hsr2.73170 (PMC13555278; doi:10.1002/hsr2.73170)
Supplement: Supplementary file 2 [file HSR2-9-e73170-s003.docx]

**Supplementary materials: 8045704**

Predictors of exclusive breastfeeding amongst mothers living with HIV in sub-Saharan Africa: a scoping review

## **Appendix I: Full list of included Sub-Saharan African countries.**

The full list of included Sub-Saharan African countries: Angola, Benin, Botswana, Burkina Faso, Burundi, Cabo Verde, Cameroon, Central African Republic, Chad, Comoros, Congo (Democratic Republic of), Congo (Republic of), Cote d’Ivoire, Equatorial Guinea, Eritrea, Eswatini (formerly known as Swaziland), Ethiopia, Gabon, Gambia, Ghana, Guinea, Guinea-Bissau, Kenya, Lesotho, Liberia, Madagascar, Malawi, Mali, Mauritania, Mauritius, Mozambique, Namibia, Niger, Nigeria, Rwanda, Sao Tome and Principe, Senegal, Seychelles, Sierra Leone, Somalia, South Africa, South Sudan, Sudan, Tanzania, Togo, Uganda, Zambia, Zimbabwe.

Subregions:

Central Africa: Angola, Burundi, Central African Republic, Chad, Congo (Democratic Republic of), Congo (Republic of), Rwanda

Eastern Africa: Comoros, Eritrea, Ethiopia, Kenya, Madagascar, Mauritius, Seychelles, Somalia, South Sudan, Sudan, Tanzania, Uganda

Southern Africa: Botswana, Eswatini (formerly known as Swaziland), Lesotho, Malawi, Mozambique, Namibia, South Africa, Zambia, Zimbabwe

Western Africa: Benin, Burkina Faso, Cabo Verde, Cameroon, Côte d’Ivoire, Equatorial Guinea, Gabon, Gambia, Ghana, Guinea, Guinea-Bissau, Liberia, Mali, Mauritania, Niger, Nigeria, São Tomé and Príncipe, Senegal, Sierra Leone, Togo

NOTE: Djibouti is not included in Sub-Saharan Africa, as it is defined by the World Bank Group as part of the Middle East and North Africa.

Supplementary material II: Inclusion and exclusion screening table

|  | **Inclusion Criteria (Include if all are met)** | ✔ | **Exclusion Criteria (Exclude if any one of these is met)** | ✔ |
| --- | --- | --- | --- | --- |
| **Population** | A focus on pregnant or breastfeeding mothers |  | Other focus populations |  |
|  | At least some of the mothers are HIV positive, and HIV status is distinguishable and incorporated into data reporting. |  | No HIV positive mothers or ambiguity on HIV status relevant to data reporting |  |
|  | Data reported by mothers, their immediate support network/ family, or healthcare professionals,  or review articles where the original literature population fulfils this |  | Data reported by other (unreliable) secondary sources |  |
| **Concept** | EBF† initiation/continuation is a measure/reported as a main outcome |  | EBF data is not measured/reported as a main outcome |  |
|  | EBF is distinct from other breastfeeding practices |  | Unclear what practices are considered EBF |  |
|  | Data from or retrospectively reporting on the EBF period (first 6 months) or prenatal EBF intentions |  | Data reported focusing on a period where EBF is not deemed necessary (after 6 months) |  |
| **Context** | Data from Sub-Saharan Africa, or one of its regions or countries |  | Data only from elsewhere |  |
|  | Studies primary data was collected from 2016 onwards. |  | Data collection before 2016 |  |
| **Study design** | Peer-reviewed journal article |  | Not a peer-reviewed journal article/ grey literature |  |
|  | Includes some quantitative data.  E.g., Experimental studies (randomized and non-randomized controlled trials, before-and-after studies, and interrupted time-series analyses), analytical observational studies (prospective and retrospective cohort studies, case-control studies, and analytical cross-sectional studies), quantitative literature reviews, or other mixed-method designs. |  | Only qualitative/low-quality data.  E.g., descriptive observational studies (case series, individual case reports, and descriptive cross-sectional studies), book chapters, opinions, commentaries, conference abstracts, websites, or policy documents. |  |
|  | Studies written in English |  | Studies in another language |  |

**Abbreviations**:

† EBF = exclusive breastfeeding

Supplementary material III: Search keywords (title, abstract, or index terms)

| **Population: HIV Positive** | **Population: Mothers** | **Concept: Exclusive Breastfeeding** | **Context: Sub-Saharan Africa** |
| --- | --- | --- | --- |
| Acquired Immunodeficiency Syndrome  HIV  Human Immunodeficiency Virus  AIDS  HIV infect*  HIV positive  HIV expos*  HIV status*  HIV transmission*  Vertical transmission  Vertical Transmission of Infections  Mother-to-child transmission*  PMTCT  Infectious disease transmission | Pregnan*  Pregnant women  Maternal health  Pregnant  Pregnant people  Gestat*  Antepartum  Postpartum  Postnatal  Matern*  Mother*  Women  Female*  Infan*  Child*  Paediatri*  pediatr*  Newborn* | Breastfeed*  Breastfeed*  Breastfed*  Breastfed*  Breast milk  Breastmilk  Exclusive breastfeed*  Exclusive breastfeed*  Formula fed*  Formular feed*  Bottle feed*  Bottle fed*  Mixed fed*  Mixed feed*  Feeding practices  Infant feed*  Infant nutrition disorders  Infant nutrition  Newborn Feed* | Africa South of the Sahara  Developing country*  Sub-Sahara* Africa*  Africa*  Low- and middle-income  LMICs  Low income  Middle income  Sub-Sahara*  South* Africa*  West* Africa*  East* Africa*  Central Africa*  Angola  Benign  Botswana  Burkina Faso  Burundi  Cabo Verde  Cameroon  Central African Republic  Chad  Comoros  Democratic Republic of Congo  Republic of Congo  Cote d’Ivoire  Equatorial Guinea  Eritrea  Eswatini  Swaziland  Ethiopia  Gabon  Gambia  Ghana  Guinea  Guinea-Bissau  Kenya  Lesotho  Liberia  Madagascar  Malawi  Mali  Mauritania  Mauritius  Mozambique  Namibia  Niger  Nigeria  Rwanda  São Tomé and Principe  Senegal  Seychelles  Sierra Leone  Somalia  South Africa  South Sudan  Sudan  Tanzania  Togo  Uganda  Zambia  Zimbabwe |

*The exact search terms used will be dependent on the MeSH/Emtree terms available, truncation, and wildcards for individual databases.

Supplementary material IV: Full database searches

## **Preliminary database searches to determine key terms**

| **Date of Search and Researcher** | **Database** | **Key Words and Search** | **Limitations on Search** | **Number of Search Results** |
| --- | --- | --- | --- | --- |
| 21.04.2025  LI | PubMed | ((HIV[Title/Abstract]) OR (Human Immunodeficiency Virus[Title/Abstract]) OR (AIDS[Title/Abstract]) OR (Acquired Immunodeficiency Syndrome[Title/Abstract]) OR (Antiretroviral therapy[Title/Abstract])) AND ((mother[Title/Abstract]) OR (pregnant[Title/Abstract]) OR (postpartum[Title/Abstract]) OR (breastfeeding[Title/Abstract]) OR (women[Title/Abstract])) AND ((breastfeeding[Title/Abstract]) OR (exclusive breastfeeding[Title/Abstract]) OR (EBF[Title/Abstract]) OR (infant feeding[Title/Abstract]) OR (breast milk[Title/Abstract]) OR (new born feeding[Title/Abstract]) OR (Prevention of mother-to-child transmission[Title/Abstract]) OR (PMTCT[Title/Abstract]) OR (vertical transmission[Title/Abstract])) AND ((Sub-Saharan Africa[Title/Abstract]) OR (Africa South of the Sahara[Title/Abstract]) OR (SSA[Title/Abstract]) OR (East Africa[Title/Abstract]) OR (West Africa[Title/Abstract]) OR (South Africa[Title/Abstract]) OR (Africa[Title/Abstract])) * | 2016-2025  Full text available  English  Study types:  Clinical Trial  Clinical Trial, Phase I  Clinical Trial, Phase II  Clinical Trial, Phase III  Clinical Trial, Phase IV  Comparative Study  Controlled Clinical Trial  Evaluation Study  Meta-Analysis  Multicentre Study  Observational Study  Pragmatic Clinical Trial  Randomized Controlled Trial  Systematic Review | 177 |
| 21.04.2025  LI | Embase (Ovid) | (HIV.mp. OR Human immunodeficiency virus/ OR AIDS.mp. OR antiretroviral therapy.mp. OR Antiretroviral Therapy/ OR Human immunodeficiency virus infection/ OR Acquired immune deficiency syndrome/) AND (mother to child transmission/ OR expectant mother/ OR adolescent mother/ OR mother.mp. OR Mother/ OR female/ OR pregnant woman/ OR pregnant.mp. OR Pregnancy/ OR postpartum.mp. OR breastfeeding.mp. OR Breast Feeding/ OR breastfeeding transmission/ OR newborn/ OR infant/ OR women.mp.) AND (breastfeeding.mp. OR Breast Feeding/ OR Breastfeeding Transmission/ OR exclusive breastfeeding.mp. OR infant feeding.mp. OR Infant Feeding/ OR breast milk.mp. OR Breast Milk/ OR newborn feeding.mp.) AND (Sub-Saharan Africa.mp. OR Africa South of the Sahara/ OR Africa south of the Sahara.mp. OR East Africa.mp. OR Africa/ OR West Africa.mp. OR South Africa.mp. OR South Africa/ OR Africa.mp.) | 2016- Current  Full text available  English  Source types: Journal | 190 |

## **Final database searches**

| **Date of Search and Researcher** | **Database** | **Key Words and Search** | **Limitations on Search** | **Number of Search Results** |
| --- | --- | --- | --- | --- |
| 22.04.2025  LI | PubMed | ("HIV Infections"[MeSH] OR "HIV Infections"[tiab] OR "Acquired Immunodeficiency Syndrome"[MeSH] OR "Acquired Immunodeficiency Syndrome"[tiab] OR HIV[tiab] OR "Human Immunodeficiency Virus"[tiab] OR AIDS[tiab] OR "HIV infect*"[tiab] OR "HIV positive"[tiab] OR "HIV expos*"[tiab] OR "HIV status*"[tiab] OR "HIV transmission*"[tiab] OR "vertical transmission"[tiab] OR "vertical transmission of infections"[MeSH] OR "mother-to-child transmission*"[tiab] OR PMTCT[tiab] OR "infectious disease transmission"[tiab])  AND  ("Pregnancy"[MeSH] OR "Pregnancy"[tiab] OR "Pregnant Women"[MeSH] OR "Pregnant Women"[tiab] OR "Maternal Health"[MeSH] OR "Maternal Health"[tiab] OR pregnan*[tiab] OR "pregnant people"[tiab] OR gestat*[tiab] OR antepartum[tiab] OR postpartum[tiab] OR postnatal[tiab] OR matern*[tiab] OR mother*[tiab] OR women[tiab] OR female*[tiab] OR "Infant"[MeSH] OR "Infant"[tiab] OR "Child"[MeSH] OR "Child"[tiab] OR "Pediatrics"[MeSH] OR "Pediatrics"[tiab] OR paediatr*[tiab] OR child*[tiab] OR infan*[tiab] OR newborn*[tiab])  AND  ("Breast Feeding"[MeSH] OR "Breast Feeding"[tiab] OR "Infant Nutrition Disorders"[MeSH] OR "Infant Nutrition"[tiab] OR breastfeed*[tiab] OR "breast feed*"[tiab] OR "breastfed"[tiab] OR "breast fed"[tiab] OR "breast milk"[tiab] OR breastmilk[tiab] OR "exclusive breastfeed*"[tiab] OR "exclusive breast feed*"[tiab] OR "formula feed*"[tiab] OR "bottle feed*"[tiab] OR "mixed feed*"[tiab] OR "formula fed*"[tiab] OR "bottle fed*"[tiab] OR "mixed fed*"[tiab] OR "feeding practice*"[tiab] OR "infant feeding"[tiab] OR "newborn feeding"[tiab])  AND  ("Africa South of the Sahara"[MeSH] OR "Africa South of the Sahara"[tiab] OR "developing Countries" [MeSH] OR "developing countr*" [tiab] OR "Sub-Saharan* Africa*"[tiab] OR Africa[tiab] OR African[tiab] OR "low- and middle-income"[tiab] OR LMIC*[tiab] OR "low-income"[tiab] OR "middle-income"[tiab] OR "Sub-Sahara"[tiab] OR "Sub-Saharan"[tiab] OR "South Africa"[tiab] OR "Southern Africa"[tiab] OR "West Africa"[tiab] OR "Western Africa"[tiab] OR "East Africa"[tiab] OR "Eastern Africa"[tiab] OR "Central Africa"[tiab] OR Angola[tiab] OR Benin[tiab] OR Botswana[tiab] OR "Burkina Faso"[tiab] OR Burundi[tiab] OR "Cabo Verde"[tiab] OR Cameroon[tiab] OR "Central African Republic"[tiab] OR Chad[tiab] OR Comoros[tiab] OR "Democratic Republic of Congo"[tiab] OR "Republic of Congo"[tiab] OR "Cote d'Ivoire"[tiab] OR "Equatorial Guinea"[tiab] OR Eritrea[tiab] OR Eswatini[tiab] OR Swaziland[tiab] OR Ethiopia[tiab] OR Gabon[tiab] OR Gambia[tiab] OR Ghana[tiab] OR Guinea[tiab] OR "Guinea-Bissau"[tiab] OR Kenya[tiab] OR Lesotho[tiab] OR Liberia[tiab] OR Madagascar[tiab] OR Malawi[tiab] OR Mali[tiab] OR Mauritania[tiab] OR Mauritius[tiab] OR Mozambique[tiab] OR Namibia[tiab] OR Niger[tiab] OR Nigeria[tiab] OR Rwanda[tiab] OR "Sao Tome and Principe"[tiab] OR Senegal[tiab] OR Seychelles[tiab] OR "Sierra Leone"[tiab] OR Somalia[tiab] OR "South Sudan"[tiab] OR Sudan[tiab] OR Tanzania[tiab] OR Togo[tiab] OR Uganda[tiab] OR Zambia[tiab] OR Zimbabwe[tiab]) | 2016-2025  Full text available  English  Study types:  Clinical Trial  Clinical Trial, Phase I  Clinical Trial, Phase II  Clinical Trial, Phase III  Clinical Trial, Phase IV  Comparative Study  Controlled Clinical Trial  Evaluation Study  Meta-Analysis  Multicentre Study  Observational Study  Pragmatic Clinical Trial  Randomized Controlled Trial  Systematic Review | 242 |
| 23.04.2025  LI | Embase (Ovid) | (exp Human immunodeficiency virus infection/ OR acquired immune deficiency syndrome.mp. OR exp Acquired immune deficiency syndrome/ OR HIV.mp. OR human immunodeficiency virus.mp. OR exp Human immunodeficiency virus/ OR AIDS.mp. OR HIV infect*.mp. OR exp Human immunodeficiency virus infected patient/ OR HIV positive.mp. OR HIV expos*.mp. OR HIV status*.mp OR HIV transmission*.mp. OR vertical transmission.mp. OR exp Vertical transmission/ OR exp Vertical transmission of infections/ OR exp Mother to child transmission/ OR mother-to-child transmission*.mp. OR PMTCT.mp. OR infectious disease transmission.mp. OR exp Disease transmission/)  AND  (exp Adolescent Pregnancy/ OR exp Pregnancy/ OR pregnant women.mp. OR exp Pregnant Woman/ OR maternal health.mp. OR pregnan*.mp. OR pregnant people.mp. OR gestat*.mp. OR antepartum.mp. OR postpartum.mp. OR exp Postnatal Care/ OR postnatal.mp. OR matern*.mp. OR exp Female/ OR exp Mother to Child Transmission/ OR exp Working Mother/ OR exp Expectant Mother/ OR exp Adolescent Mother/ OR exp Mother Child Relation/ OR mother*.mp. OR exp Mother/ OR women.mp. OR female*.mp. OR exp Infant/ OR infan*.mp. OR newborn*.mp. OR exp Newborn/ OR exp Newborn Care/ OR exp Child/ OR exp Child Nutrition/ OR exp Child Health/ OR exp Pediatrics/ OR paediatr*.mp. OR pediatr*.mp. OR child*.mp.)  AND  (breastfeeding.mp. OR exp Breast Feeding/ OR infant nutrition disorders.mp. OR infant nutrition.mp. OR exp Infant Nutrition/ OR breastfeed*.mp. OR exp Breast Feeding Education/ OR exp Breast Milk/ OR breast feed*.mp. OR breastfed*.mp. OR breastfed. OR breast milk.mp. OR breastmilk.mp. OR exclusive breastfeed*.mp. OR exclusive breast feed*.mp. OR exp Infant Feeding/ OR formula feed*.mp. OR exp Bottle Feeding/ OR bottle feed*.mp. OR mixed feed*.mp. OR formula fed*.mp. OR bottle fed*.mp. OR mixed fed*.mp. OR exp Feeding Behavior/ OR feeding practice*.mp. OR infant feed*.mp. OR newborn feed*.mp.)  AND  ("Africa south of the Sahara".mp. OR exp Africa South of the Sahara OR developing countries. OR exp Developing Country/ OR "Sub-Saharan Africa*".mp. OR Africa*.mp. OR exp Africa/ OR exp African/ OR exp Sub-Saharan African/ OR exp Central African/ OR exp Southern African/ OR exp South African/ OR exp West African/ OR exp East African/ OR exp Central Africa/ OR exp Middle Income Country/ OR exp Low Income Country/ OR "low- and middle-income".mp. OR LMIC*.mp. OR low-income.mp. OR middle-income.mp. OR Sub-Sahara*.mp. OR "South* Africa*".mp. OR East* Africa*.mp. OR West* Africa*.mp. OR Central Africa*.mp.  OR  Angola.mp. OR Benign.mp. OR Botswana.mp. OR Burkina Faso.mp. OR Burundi.mp. OR Cabo Verde.mp. OR Cameroon.mp. OR Central African Republic.mp. OR Chad.mp. OR Comoros.mp. OR Democratic Republic of Congo.mp. OR Republic of Congo.mp. OR Côte d’Ivoire.mp. OR Equatorial Guinea.mp. OR Eritrea.mp. OR Eswatini.mp. OR Swaziland.mp. OR Ethiopia.mp. OR Gabon.mp. OR Gambia.mp. OR Ghana.mp. OR Guinea.mp. OR Guinea-Bissau.mp. OR Kenya.mp. OR Lesotho.mp. OR Liberia.mp. OR Madagascar.mp. OR Malawi.mp. OR Mali.mp. OR Mauritania.mp. OR Mauritius.mp. OR Mozambique.mp. OR Namibia.mp. OR Niger.mp. OR Nigeria.mp. OR Rwanda.mp. OR "Sao Tome and Principe".mp. OR Senegal.mp. OR Seychelles.mp. OR Sierra Leone.mp. OR Somalia.mp. OR South Africa.mp. OR South Sudan.mp. OR Sudan.mp. OR Tanzania.mp. OR Togo.mp. OR Uganda.mp. OR Zambia.mp. OR Zimbabwe.mp.  OR  exp Angola/ OR exp Benin/ OR exp Botswana/ OR exp Burkina Faso/ OR exp Burundi/ OR exp Cabo Verde/ OR exp Cameroon/ OR exp Central African Republic/ OR exp Chad/ OR exp Comoros/ OR exp Democratic Republic of Congo/ OR exp Republic of Congo/ OR exp Equatorial Guinea/ OR exp Eritrea/ OR exp Eswatini/ OR exp Swaziland/ OR exp Ethiopia/ OR exp Gabon/ OR exp Gambia/ OR exp Ghana/ OR exp Guinea/ OR exp Guinea-Bissau/ OR exp Kenya/ OR exp Lesotho/ OR exp Liberia/ OR exp Madagascar/ OR exp Malawi/ OR exp Mali/ OR exp Mauritania/ OR exp Mauritius/ OR exp Mozambique/ OR exp Namibia/ OR exp Niger/ OR exp Nigeria/ OR exp Rwanda/ OR exp "Sao Tome and Principe"/ OR exp Senegal/ OR exp Seychelles/ OR exp Sierra Leone/ OR exp Somalia/ OR exp South Africa/ OR exp South Sudan/ OR exp Sudan/ OR exp Tanzania/ OR exp Togo/ OR exp Uganda/ OR exp Zambia/ OR exp Zimbabwe/) | 2016- Current  Full text available  English  Embase  Source types: Journal | 320 |
| 23.04.2025  LI | Web of Science Core Collection | (TI=(HIV OR "Human Immunodeficiency Virus" OR "Acquired Immunodeficiency Syndrome" OR "HIV infect*" OR "HIV positive" OR "HIV expos*" OR "HIV status*" OR "HIV transmission*" OR "Vertical transmission" OR "Vertical transmission of infections" OR "Mother-to-child transmission" OR "PMTCT" OR "Infectious disease transmission")  OR AB=(HIV OR "Human Immunodeficiency Virus" OR "Acquired Immunodeficiency Syndrome" OR "HIV infect*" OR "HIV positive" OR "HIV expos*" OR "HIV status*" OR "HIV transmission*" OR "Vertical transmission" OR "Vertical transmission of infections" OR "Mother-to-child transmission" OR "PMTCT" OR "Infectious disease transmission"))  AND  (TI=("Pregnan*" OR "Pregnant women" OR "Maternal health" OR "Pregnant" OR "Pregnant people" OR "Gestat*" OR "Antepartum" OR "Postpartum" OR "Postnatal" OR "Matern*" OR "Mother*" OR "Women" OR "Female*" OR "Infan*" OR "Child*" OR "Paediatri*" OR "Pediatr*" OR "Newborn*")  OR AB=("Pregnan*" OR "Pregnant women" OR "Maternal health" OR "Pregnant" OR "Pregnant people" OR "Gestat*" OR "Antepartum" OR "Postpartum" OR "Postnatal" OR "Matern*" OR "Mother*" OR "Women" OR "Female*" OR "Infan*" OR "Child*" OR "Paediatri*" OR "Pediatr*" OR "Newborn*"))  AND  (TI=("Breastfeed*" OR "Breast feed*" OR "Breastfed*" OR "Breast fed*" OR "Breast milk" OR "Breastmilk" OR "Exclusive breastfeed*" OR "Exclusive breast feed*" OR "Formula fed*" OR "Formula feed*" OR "Bottle feed*" OR "Bottle fed*" OR "Mixed fed*" OR "Mixed feed*" OR "Feeding practices" OR "Infant feed*" OR "Infant nutrition disorders" OR "Infant nutrition" OR "Newborn Feed*")  OR AB=("Breastfeed*" OR "Breast feed*" OR "Breastfed*" OR "Breast fed*" OR "Breast milk" OR "Breastmilk" OR "Exclusive breastfeed*" OR "Exclusive breast feed*" OR "Formula fed*" OR "Formula feed*" OR "Bottle feed*" OR "Bottle fed*" OR "Mixed fed*" OR "Mixed feed*" OR "Feeding practices" OR "Infant feed*" OR "Infant nutrition disorders" OR "Infant nutrition" OR "Newborn Feed*"))  AND  (TI=("Africa South of the Sahara" OR "Developing countr*" OR "Sub-Sahara* Africa*" OR "Africa*" OR "Low- and middle- income" OR "LMICs" OR "Low income" OR "Middle income" OR "Sub-Sahara*" OR "South* Africa*" OR "West* Africa*" OR "East* Africa*" OR "Central Africa*" OR "Angola" OR "Benin" OR "Botswana" OR "Burkina Faso" OR "Burundi" OR "Cabo Verde" OR "Cameroon" OR "Central African republic" OR "Chad" OR "Comoros" OR "Democratic Republic of Congo" OR "Republic of Congo" OR "Cote d’Ivoire" OR "Equatorial Guinea" OR "Eritrea" OR "Eswatini" OR "Swaziland" OR "Ethiopia" OR "Gabon" OR "Gambia" OR "Ghana" OR "Guinea" OR "Guinea-Bissau" OR "Kenya" OR "Lesotho" OR "Liberia" OR "Madagascar" OR "Malawi" OR "Mali" OR "Mauritania" OR "Mauritius" OR "Mozambique" OR "Namibia" OR "Niger" OR "Nigeria" OR "Rwanda" OR "Sao Tome and Principe" OR "Senegal" OR "Seychelles" OR "Sierra Leone" OR "Somalia" OR "South Africa" OR "South Sudan" OR "Sudan" OR "Tanzania" OR "Togo" OR "Uganda" OR "Zambia" OR "Zimbabwe")  OR AB=("Africa South of the Sahara" OR "Developing countr*" OR "Sub-Sahara* Africa*" OR "Africa*" OR "Low- and middle- income" OR "LMICs" OR "Low income" OR "Middle income" OR "Sub-Sahara*" OR "South* Africa*" OR "West* Africa*" OR "East* Africa*" OR "Central Africa*" OR "Angola" OR "Benin" OR "Botswana" OR "Burkina Faso" OR "Burundi" OR "Cabo Verde" OR "Cameroon" OR "Central African republic" OR "Chad" OR "Comoros" OR "Democratic Republic of Congo" OR "Republic of Congo" OR "Cote d’Ivoire" OR "Equatorial Guinea" OR "Eritrea" OR "Eswatini" OR "Swaziland" OR "Ethiopia" OR "Gabon" OR "Gambia" OR "Ghana" OR "Guinea" OR "Guinea-Bissau" OR "Kenya" OR "Lesotho" OR "Liberia" OR "Madagascar" OR "Malawi" OR "Mali" OR "Mauritania" OR "Mauritius" OR "Mozambique" OR "Namibia" OR "Niger" OR "Nigeria" OR "Rwanda" OR "Sao Tome and Principe" OR "Senegal" OR "Seychelles" OR "Sierra Leone" OR "Somalia" OR "South Africa" OR "South Sudan" OR "Sudan" OR "Tanzania" OR "Togo" OR "Uganda" OR "Zambia" OR "Zimbabwe"))  <https://www.webofscience.com/wos/woscc/summary/07bb414a-e92e-4f32-922f-e6af548a5f74-015d40528e/relevance/1>  ^ Search link | 2016-2025  English  Study types:  Articles  Review Articles | 878 |
| 09.05.2025  LI | Scopus (Elsevier) | ( TITLE-ABS-KEY ( "Acquired Immunodeficiency Syndrome" OR hiv OR "Human Immunodeficiency Virus" OR aids OR "HIV infect*" OR "HIV positive" OR "HIV expos*" OR "HIV status*" OR "HIV transmission*" OR "Vertical transmission" OR "Vertical transmission of infections" OR "Mother-to-child transmission*" OR pmtct OR "infectious disease transmission" ) AND TITLE-ABS-KEY ( pregnan* OR "pregnant women" OR "maternal health" OR pregnant OR "pregnant people" OR gestat* OR antepartum OR postpartum OR postnatal OR matern* OR mother* OR women OR female* OR infan* OR child* OR paediatri* OR pediatr* OR newborn* ) AND TITLE-ABS-KEY ( breastfeed* OR "breast feed*" OR breastfed* OR "breast fed*" OR "breast milk" OR breastmilk OR "exclusive breastfeed*" OR "exclusive breast feed*" OR "formula fed*" OR "formular feed*" OR "bottle feed*" OR "bottle fed*" OR "mixed fed*" OR "mixed feed*" OR "feeding practices" OR "infant feed*" OR "infant nutrition disorders" OR "infant nutrition" OR "newborn feed*" ) AND TITLE-ABS-KEY ( "Africa South of the Sahara" OR "developing countr*" OR "Sub-Sahara* Africa*" OR africa* OR "low- and middle-income" OR lmic* OR "low income" OR "middle income" OR "Sub-Sahara*" OR "South* Africa*" OR "West* Africa*" OR "East* Africa*" OR "Central Africa*" OR angola OR benign OR botswana OR "Burkina Faso" OR burundi OR "Cabo Verde" OR cameroon OR "Central African Republic" OR chad OR comoros OR "Democratic Republic of Congo" OR "Republic of Congo" OR "Cote d&apos;Ivoire" OR "Equatorial Guinea" OR eritrea OR eswatini OR swaziland OR ethiopia OR gabon OR gambia OR ghana OR guinea OR "Guinea-Bissau" OR kenya OR lesotho OR liberia OR madagascar OR malawi OR mali OR mauritania OR mauritius OR mozambique OR namibia OR niger OR nigeria OR rwanda OR "Sao Tome and Principe" OR senegal OR seychelles OR "Sierra Leone" OR somalia OR "South Africa" OR "South Sudan" OR sudan OR tanzania OR togo OR uganda OR zambia OR zimbabwe ) )  AND PUBYEAR > 2015 AND PUBYEAR < 2026 AND ( LIMIT-TO ( DOCTYPE , "ar" ) OR LIMIT-TO ( DOCTYPE , "re" ) ) AND ( LIMIT-TO ( LANGUAGE , "English" ) ) AND ( LIMIT-TO ( SRCTYPE , "j" ) ) | 2016- Current  Study types:  Article Review Articles  English  Source types: Journal | 1279 |
| 09.05.2025  LI | Global Health (Ovid) | (exp HIV infections/ OR "acquired immune deficiency syndrome".mp. OR exp AIDS/ OR HIV.mp. OR "human immunodeficiency virus".mp. OR AIDS.mp. OR "HIV infect*".mp. OR "HIV positive".mp. OR "HIV expos*".mp. OR "HIV status*".mp. OR "HIV transmission*".mp. OR "vertical transmission".mp. OR exp Vertical transmission/ OR exp Mother to child transmission/ OR "mother-to-child transmission*".mp. OR PMTCT.mp. OR "infectious disease transmission".mp. OR exp Disease transmission/)  AND  (exp Pregnancy/ OR "pregnant women".mp. OR exp Pregnant woman/ OR "maternal health".mp. OR pregnan*.mp. OR "pregnant people".mp. OR gestat*.mp. OR antepartum.mp. OR postpartum.mp. OR exp Postnatal care/ OR postnatal.mp. OR matern*.mp. OR exp Female/ OR exp Mother to child transmission/ OR exp Working mother/ OR exp Expectant mother/ OR exp Adolescent mother/ OR exp Mother child relation/ OR mother*.mp. OR exp Mother/ OR women.mp. OR female*.mp. OR exp Infant/ OR infan*.mp. OR newborn*.mp. OR exp Newborn/ OR exp Newborn care/ OR exp Child/ OR exp Child nutrition/ OR exp Child health/ OR exp Pediatrics/ OR paediatr*.mp. OR pediatr*.mp. OR child*.mp.)  AND  ("breastfeeding".mp. OR exp Breastfeeding/ OR "infant nutrition disorders".mp. OR "infant nutrition".mp. OR exp Infant nutrition/ OR breastfeed*.mp. OR exp Breastfeeding education/ OR exp Breast milk/ OR "breast feed*".mp. OR breastfed*.mp. OR "breastfed*".mp. OR "breast milk".mp. OR breastmilk.mp. OR "exclusive breastfeed*".mp. OR "exclusive breast feed*".mp. OR exp Infant feeding/ OR "formula feed*".mp. OR exp Bottle feeding/ OR "bottle feed*".mp. OR "mixed feed*".mp. OR "formula fed*".mp. OR "bottle fed*".mp. OR "mixed fed*".mp. OR exp Feeding behavior/ OR "feeding practice*".mp. OR "infant feed*".mp. OR "newborn feed*".mp.)  AND  ("Africa south of the Sahara".mp. OR exp Africa South of the Sahara/ OR "developing country ".mp. OR exp Developing countries/ OR "Sub-Saharan Africa*".mp. OR Africa*.mp. OR exp Africa/ OR exp African/ OR exp Sub-Saharan Africa/ OR exp Central Africa/ OR exp Southern Africa/ OR exp South Africa/ OR exp West Africa/ OR exp East Africa/ OR exp Middle income countries/ OR exp Low income countries/ OR "low- and middle-income".mp. OR LMIC*.mp. OR "low-income".mp. OR "middle-income".mp. OR Sub-Sahara*.mp. OR "South* Africa*".mp. OR East* Africa*.mp. OR West* Africa*.mp. OR Central Africa*.mp.  OR  Angola.mp. OR Bénin.mp. OR Botswana.mp. OR Burkina Faso.mp. OR Burundi.mp. OR Cabo Verde.mp. OR Cameroon.mp. OR Central African Republic.mp. OR Chad.mp. OR Comoros.mp. OR Democratic Republic of Congo.mp. OR Republic of Congo.mp. OR Côte d’Ivoire.mp. OR Equatorial Guinea.mp. OR Eritrea.mp. OR Eswatini.mp. OR Swaziland.mp. OR Ethiopia.mp. OR Gabon.mp. OR Gambia.mp. OR Ghana.mp. OR Guinea.mp. OR Guinea-Bissau.mp. OR Kenya.mp. OR Lesotho.mp. OR Liberia.mp. OR Madagascar.mp. OR Malawi.mp. OR Mali.mp. OR Mauritania.mp. OR Mauritius.mp. OR Mozambique.mp. OR Namibia.mp. OR Niger.mp. OR Nigeria.mp. OR Rwanda.mp. OR "Sao Tome and Principe".mp. OR Senegal.mp. OR Seychelles.mp. OR Sierra Leone.mp. OR Somalia.mp. OR South Africa.mp. OR South Sudan.mp. OR Sudan.mp. OR Tanzania.mp. OR Togo.mp. OR Uganda.mp. OR Zambia.mp. OR Zimbabwe.mp.  OR  exp Angola/ OR exp Benin/ OR exp Botswana/ OR exp Burkina Faso/ OR exp Burundi/ OR exp Cabo Verde/ OR exp Cameroon/ OR exp Central African Republic/ OR exp Chad/ OR exp Comoros/ OR exp Democratic Republic of Congo/ OR exp Republic of Congo/ OR exp Equatorial Guinea/ OR exp Eritrea/ OR exp Eswatini/ OR exp Swaziland/ OR exp Ethiopia/ OR exp Gabon/ OR exp Gambia/ OR exp Ghana/ OR exp Guinea/ OR exp Guinea-Bissau/ OR exp Kenya/ OR exp Lesotho/ OR exp Liberia/ OR exp Madagascar/ OR exp Malawi/ OR exp Mali/ OR exp Mauritania/ OR exp Mauritius/ OR exp Mozambique/ OR exp Namibia/ OR exp Niger/ OR exp Nigeria/ OR exp Rwanda/ OR exp Sao Tome and Principe/ OR exp Senegal/ OR exp Seychelles/ OR exp Sierra Leone/ OR exp Somalia/ OR exp South Africa/ OR exp South Sudan/ OR exp Sudan/ OR exp Tanzania/ OR exp Togo/ OR exp Uganda/ OR exp Zambia/ OR exp Zimbabwe/) | 2016- Current  Full text available  English  Global Health  Source types: Journal | 217 |

Supplementary material VI: Study Design Appraisal

| **Study** | **Reviewer 1 (LI) – Study design** | **Reviewer 2 (XJ) – Study design** |
| --- | --- | --- |
| Dagnew 2019 | A quantitative cross-sectional study | A quantitative cross-sectional study |
| Iliyasu 2019 | A quantitative cross-sectional study | A quantitative cross-sectional study |
| Mekonnen 2022 | A quantitative cross-sectional study | A quantitative cross-sectional study |
| Mebratu 2020 | A quantitative cross-sectional study | A quantitative cross-sectional study |
| Napyo 2020 | A prospective cohort study | A prospective cohort study |
| Faustine 2022 | A quantitative cross-sectional study | A quantitative cross-sectional study |
| Zewdu 2023 | A quantitative cross-sectional study | A quantitative cross-sectional study |
| Zunza M 2023 | Randomised control feasibility trial (type of experimental study) | An experimental study |
| Mukhula 2025 | Secondary analysis of a cross-sectional study | Secondary data analysis of a cross-sectional study |
| Muhammed 2019 | A case-control study | A case-control study |
| Goon 2021 | A quantitative cross-sectional study | A quantitative cross-sectional study |
| Etowa 2021 | A quantitative cross-sectional study | A quantitative cross-sectional study |
| Genetu 2017 | A quantitative cross-sectional study | A quantitative cross-sectional study |
| Bultum 2022 | A quantitative cross-sectional study | A quantitative cross-sectional study |
| Zunza M 2025 | Randomised control trial (type of experimental study) | An experimental study |
| Mutawulira 2022 | Mixed methods cross-sectional study | A quantitative cross-sectional study |
| Gejo 2019 | A quantitative cross-sectional study | A quantitative cross-sectional study |
| Philemon 2022 | A quantitative cross-sectional study | A quantitative cross-sectional study |
| Ejara 2018 | A quantitative cross-sectional study | A quantitative cross-sectional study |

Supplementary material VII: Results by predictor table

| **Predictor variable** | **Positive predictor of EBF (reference, study design, bivariate (B) or multivariate (M) analysis)** | **Negative predictor of EBF (reference, study design, bivariate (B) or multivariate (M) analysis)** |
| --- | --- | --- |
| Antenatal care  8 | (Dagnew et al., 2019) -Quantitative cross-sectional study (M)  (Mebratu et al., 2020) - Quantitative cross-sectional study (M)  (Faustine & Moshi, 2022) - Quantitative cross-sectional study (M)  (Etowa et al., 2021) - Quantitative cross-sectional study(M)  (Ejara et al., 2018) - Quantitative cross-sectional study (M) | (Mekonnen et al., 2022) - Quantitative cross-sectional study (M)  (Mukhula et al., 2025) - Secondary analysis of a cross-sectional study (B)  (Philemon et al., 2022) - Quantitative cross-sectional study (M) |
| Disclosure of HIV status  6 | (Dagnew et al., 2019) - Quantitative cross-sectional study (M)  (Mebratu et al., 2020) - Quantitative cross-sectional study (M)  (Zewdu et al., 2023) - Quantitative cross-sectional study (M)  (Bultum et al., 2022) - Quantitative cross-sectional study (M)  (Ejara et al., 2018) - Quantitative cross-sectional study (M) | (Mekonnen et al., 2022) - Quantitative cross-sectional study (M) |
| Good knowledge  4 | (Mebratu et al., 2020) - Quantitative cross-sectional study (M)  (Faustine & Moshi, 2022) - Quantitative cross-sectional study (M)  (Bultum et al., 2022) - Quantitative cross-sectional study (M)  (Gejo et al., 2019) - Quantitative cross-sectional study (M) |  |
| Infant feeding counselling  5 | (Iliyasu et al., 2019) - Quantitative cross-sectional study (M)  (Genetu et al., 2016) - Quantitative cross-sectional study (M)  (Bultum et al., 2022) - Quantitative cross-sectional study (M)  (Gejo et al., 2019) - Quantitative cross-sectional study (M)  (Ejara et al., 2018) - Quantitative cross-sectional study (M) |  |
| Favourable attitudes  5 | (Mebratu et al., 2020) - Quantitative cross-sectional study (M)  (Faustine & Moshi, 2022) - Quantitative cross-sectional study (M)  (Gejo et al., 2019) - Quantitative cross-sectional study (M)  (Ejara et al., 2018) - Quantitative cross-sectional study (M)  (Etowa et al., 2021) - Quantitative cross-sectional study(M) |  |
| Hospital delivery  4 | (Iliyasu et al., 2019) - Quantitative cross-sectional study (M)  (Mutawulira et al., 2022) - Mixed methods cross-sectional study (M) | (Mekonnen et al., 2022) - Quantitative cross-sectional study (M)  (Muhammed & Seid, 2019) - Unmatched case control study (M) |
| Higher income  3 | (Faustine & Moshi, 2022) - Quantitative cross-sectional study (M)  (Mutawulira et al., 2022) - Mixed methods cross-sectional study (M) | (Napyo et al., 2020) - Quantitative prospective cohort study (M) |
| Postnatal care  3 | (Dagnew et al., 2019) - Quantitative cross-sectional study (M)  (Zewdu et al., 2023) - Quantitative cross-sectional study (M)  (Ejara et al., 2018) - Quantitative cross-sectional study (M) |  |
| Education  3 | (Dagnew et al., 2019) - Quantitative cross-sectional study (M) | (Muhammed & Seid, 2019) - Unmatched case control study (M)  (Goon et al., 2021) - Quantitative cross-sectional study (M) |
| ART adherence  2 | (Napyo et al., 2020) - Quantitative prospective cohort study (M)  (Zewdu et al., 2023) - Quantitative cross-sectional study (M) |  |
| Breast problems  2 |  | (Faustine & Moshi, 2022) - Quantitative cross-sectional study (M)  (Ejara et al., 2018) - Quantitative cross-sectional study (M) |
| Employment  2 |  | (Muhammed & Seid, 2019) - Unmatched case control study (M)  (Goon et al., 2021) - Quantitative cross-sectional study (M) |
| Pregnancy complications  2 |  | (Mekonnen et al., 2022) - Quantitative cross-sectional study (M)  (Genetu et al., 2016) - Quantitative cross-sectional study (M) |
| Social support  2 | (Mekonnen et al., 2022) - Quantitative cross-sectional study (M)  (Etowa et al., 2021) - Quantitative cross-sectional study (M) |  |
| More than one child  1 | (Philemon et al., 2022) - Quantitative cross-sectional study (M) |  |
| Delivery supervised by health worker  1 | (Napyo et al., 2020) - Quantitative prospective cohort study (M) |  |
| HIV positive for less than 5 years  1 | (Philemon et al., 2022) - Quantitative cross-sectional study (M) |  |
| Older maternal age  1 |  | (Mekonnen et al., 2022) - Quantitative cross-sectional study (M) |
| Urban residence  1 |  | (Mekonnen et al., 2022) - Quantitative cross-sectional study (M) |
| Alcohol consumption  1 |  | (Goon et al., 2021) - Quantitative cross-sectional study (M) |
| Frequent early infant diagnosis clinic attendance  1 |  | (Mutawulira et al., 2022) - Mixed methods cross-sectional study (M) |
| Increased child age  1 |  | (Mutawulira et al., 2022) - Mixed methods cross-sectional study (M) |
| Infant mouth ulcers  1 |  | (Ejara et al., 2018) - Quantitative cross-sectional study (M) |
| Stress  1 |  | (Etowa et al., 2021) - Quantitative cross-sectional study (M) |

Supplementary material VIII: Summary of predictors of exclusive breastfeeding (EBF) or recommended feeding practices identified in included studies

**Predictors positively or negatively associated with EBF or EBF/ERF in the included studies**

| **Predictor**  **(Comparison vs Reference)** | **Study** | **Population** | **Analysis** | **Outcome**  **vs no outcome** | **Effect estimate (95% CI)** | **p-value*** | **Direction of association between predictor and EBF or EBF/ERF** | **Covariates included in adjusted model** |
| --- | --- | --- | --- | --- | --- | --- | --- | --- |
| Antenatal care | | | | | | | | |
| Antenatal care follow-up  (Yes vs No) | Dagnew et al., 2019 | N = 213  HIV-positive mothers | Multivariate logistic regression | EBF/ERF vs MF | AOR = 6.7 (5.6-10.9) | P < 0.05 | Positive | Variables with P < 0.05 in bivariable analysis; exact covariates not reported. |
| Antenatal care follow-up  (Yes vs No) | Mekonnen et al., 2022 | N = 355 HIV-positive mothers | Multivariate logistic regression | EBF/ERF vs MF | AOR = 0.003  (0.00-0.03) | P < 0.05 | Negative | Variables with P < 0.20 in bivariable analysis; exact covariates not reported. |
| Antenatal care (Yes vs No) | Mebratu et al., 2020 | N = 209 HIV-positive mothers | Multivariate logistic regression | EBF vs non-EBF | AOR = 3.01  (1.1-8.28) | P < 0.05 | Positive | Variables with p < 0.25 in bivariable analysis; exact covariates not reported. |
| Antenatal care (Adequate vs Inadequate) | Faustine & Moshi, 2022 | N = 372 HIV-positive mothers | Multivariate logistic regression | EBF vs non-EBF | AOR = 1.76 (1.09-2.82) | P = 0.002 | Positive | Variables with P < 0.05 in bivariable analysis; exact covariates not reported. |
| Antenatal care  (Yes vs No) | Etowa et al., 2021 | N = 690  HIV-positive women | Multinomial logistic regression | EBF vs MF | RRR = 20.2 | P < 0.05 | Positive | National policy guideline, maternal age, marital status, number of children born after HIV diagnosis, maternal education, maternal employment, baby's father's attitude, family’s attitude, cultural beliefs, source of prenatal care, functional social support, perceived stress, infant feeding attitude |
| >= 1 Antenatal care visit during the pregnancy  (Yes vs No) | Ejara at al., 2018 | N = 283  HIV-positive mothers | Multivariate logistic regression | Non-EBF vs EBF*** | AOR = 0.05 (0.01-0.30) | P < 0.05 | Positive | Attitude on EBF, mother’s breast problem, infant had mouth ulcers, disclosure of HIV status, maternal education, place of delivery, postnatal care, and infant feeding counselling. |
| More antenatal visits  (5-12 vs 0-4) | Mukhula et al., 2025 | N = 771 mother-infant pairs  (n = 143  HIV-exposed infants aged 0-6 months) | Bivariate log-binomial regression | EBF vs non-EBF | Prevalence Ratio = 0.62 (0.41-0.93) | P < 0.05 | Negative | 0 |
| More antenatal visits  (>= 4 vs < 4) | Philemon et al., 2022 | N = 524 mother-infant pairs | Multivariate logistic regression | EBF vs non-EBF | AOR = 0.41 (0.23-0.74) | P = 0.003 | Negative | Exact covariates not reported. |
| Disclosure of HIV status | | | | | | | | |
| Disclosure of HIV status  (Yes vs No) | Dagnew et al., 2019 | N = 213  HIV-positive mothers | Multivariate logistic regression | EBF/ERF vs MF | AOR = 8.2  (6.2-10.7) | P < 0.05 | Positive | Variables with P < 0.05 in bivariable analysis; exact covariates not reported. |
| Disclosure of HIV status  (No vs Yes)** | Mekonnen et al., 2022 | N = 355 HIV-positive mothers | Multivariate logistic regression | EBF/ERF vs MF | AOR = 3.17 (1.12-8.99) | P < 0.05 | Negative | Variables with P < 0.20 in bivariable analysis; exact covariates not reported. |
| Disclosure of HIV status  (Yes vs No) | Mebratu et al., 2020 | N = 209 HIV-positive mothers | Multivariate logistic regression | EBF vs non-EBF | AOR = 3.17 (1.12-8.99) | P < 0.05 | Positive | Variables with p < 0.25 in bivariable analysis; exact covariates not reported. |
| Disclosure of HIV status  (No vs Yes)** | Zewdu et al., 2023 | N = 423 HIV-positive mothers | Multivariate logistic regression | MF vs EBF*** | AOR = 2.32 (1.47-3.67) | P = 0.001 | Positive | Variables with p < 0.25 in bivariable analysis; exact covariates not reported. |
| Disclosure of HIV status  (Yes vs No) | Bultum et al., 2022 | N = 219 HIV-positive mothers | Multivariate logistic regression | EBF vs non-EBF | AOR = 6.0 (1.2-29.6) | P < 0.05 | Positive | Variables with p <= 0.05 in bivariable analysis; exact covariates are not reported. |
| Disclosure of HIV status  (Yes vs No) | Ejara et al., 2018 | N = 283  HIV-positive mothers | Multivariate logistic regression | Non-EBF vs EBF*** | AOR = 0.28 (0.12-0.63) | P < 0.05 | Positive | Attitude on EBF, mother’s breast problem, infant having mouth ulcers, maternal education, place of delivery, postnatal care, infant feeding counselling, and antenatal care. |
| Good knowledge | | | | | | | | |
| Good knowledge on IFP  (Sufficient vs. Insufficient) | Mebratu et al., 2020 | N = 209 HIV-positive mothers | Multivariate logistic regression | EBF vs non-EBF | AOR = 3.32 (1.15-9.55) | P < 0.05 | Positive | Variables with p < 0.25 in bivariable analysis; exact covariates not reported. |
| Good knowledge on EBF (Adequate vs Inadequate) | Faustine & Moshi, 2022 | N = 372 HIV-positive mothers | Multivariate logistic regression | EBF vs non-EBF | AOR = 5.11  (3.20-8.17) | P < 0.001 | Positive | Variables with P < 0.05 in bivariable analysis; exact covariates not reported. |
| Known HIV transmitted during delivery  (Yes vs don’t know) | Bultum et al., 2022 | N = 219 HIV-positive mothers | Multivariate logistic regression | EBF vs non-EBF | AOR = 5.2 (1.1-24.4) | P < 0.05 | Positive | Variables with p <= 0.05 in bivariable analysis; exact covariates not reported. |
| Good knowledge on EBF  (Good vs Poor) | Gejo et al., 2019 | N = 304 HIV-positive mothers | Multivariate logistic regression | EBF vs non-EBF | AOR = 5.45 (2.12-14.02) | P < 0.05 | Positive | Maternal education, marital status, monthly income, employment, place of residence, number of children, HIV disclosure status, antenatal care, mode of delivery, attitude towards EBF, infant feeding counselling. |
| Infant feeding counselling | | | | | | | | |
| Received counselling on infant feeding  (Yes vs No) | Iliyasu et al., 2019 | N = 203  HIV-positive mothers | Multivariate logistic regression | EBF vs non-EBF | AOR = 2.16 (1.28-4.15) | P = 0.02 | Positive | Maternal age, occupation, place of delivery, knowledge of appropriate feeding. |
| EBF counseling (Yes vs No) | Genetu et al., 2016 | N = 367  Mother-child pairs | Multivariate logistic regression | EBF vs non-EBF | AOR = 5.1 (1.4-18.2) | P < 0.05 | Positive | Variables with P < 0.20 in bivariable analysis; exact covariates not reported |
| Received IFP advice from health workers  (Yes vs No) | Bultum et al., 2022 | N = 219 HIV-positive mothers | Multivariate logistic regression | EBF vs non-EBF | AOR = 3.0  (1.2-6.7) | P < 0.05 | Positive | Variables with p <= 0.05 in bivariable analysis; exact covariates not reported. |
| Infant feeding counselling  (Yes vs No) | Gejo et al., 2019 | N = 304 HIV-positive mothers | Multivariate logistic regression | EBF vs non-EBF | AOR = 6.87 (2.63-4.02) | P < 0.05 | Positive | Maternal education, marital status, monthly income, employment, place of residence, number of children, HIV disclosure status, antenatal care, mode of delivery, attitude towards EBF, knowledge on EBF. |
| Infant feeding counselling  (Yes vs No) | Ejara et al., 2018 | N = 283  HIV-positive mothers | Multivariate logistic regression | Non-EBF vs EBF*** | AOR = 0.18 (0.06-0.55) | P < 0.05 | Positive | Attitude on EBF, mother’s breast problem, infant had mouth ulcers, disclosure of HIV status, maternal education, place of delivery, postnatal care, antenatal care. |
| Favourable attitudes |  |  |  |  |  |  |  |  |
| Favourable attitudes towards IFP (Favourable vs. Unfavourable) | Mebratu et al., 2020 | N = 209 HIV-positive mothers | Multivariate logistic regression | EBF vs non-EBF | AOR = 5.39 (1.65-17.6) | P < 0.05 | Positive | Variables with p < 0.25 in bivariable analysis; exact covariates not reported. |
| Perceptions of EBF  (Positive vs Negative) | Faustine & Moshi, 2022 | N = 372 HIV-positive mothers | Multivariate logistic regression | EBF vs non-EBF | AOR = 3.51  (2.25-5.47) | P < 0.001 | Positive | Variables with P < 0.05 in bivariable analysis; exact covariates not reported. |
| Favourable attitudes towards EBF (Favourable vs Unfavourable) | Gejo et al., 2019 | N = 304 HIV-positive mothers | Multivariate logistic regression | EBF vs non-EBF | AOR = 7.92 (2.96-21.21) | P < 0.05 | Positive | Maternal education, marital status, monthly income, employment, place of residence, number of children, HIV disclosure status, antenatal care, mode of delivery, knowledge on EBF, infant feeding counselling. |
| Favourable attitudes towards IFP (Favourable vs. Unfavourable) | Ejara et al., 2018 | N = 283  HIV-positive mothers | Multivariate logistic regression | Non-EBF vs EBF*** | AOR = 0.39 (0.16-0.97) | P < 0.05 | Positive | Mother’s breast problem, infant had mouth ulcers, disclosure of HIV status, maternal education, place of delivery, postnatal care, infant feeding counselling, antenatal care. |
| Favourable attitudes towards IFP (score on Iowa scale) | Etowa et al., 2021 | N = 690  HIV-positive women | Multinomial logistic regression | EBF vs MF | RRR = 1.1 | P < 0.05 | Positive | National policy guideline, maternal age, marital status, number of children born after HIV diagnosis, maternal education, maternal employment, baby's father attitude, family’s attitude, cultural beliefs, antenatal care, social support, source of prenatal care, perceived stress. |
| Hospital delivery |  |  |  |  |  |  |  |  |
| Hospital delivery  (Hospital vs Home) | Iliyasu et al., 2019 | N = 203  HIV-positive mothers | Multivariate logistic regression | EBF vs non-EBF | AOR = 3.02 (2.67-7.84) | P = 0.03 | Positive | Maternal age, occupation, IFP counseling, knowledge of appropriate feeding. |
| Hospital delivery  (Home vs Health facility)** | Muhammed & Seid, 2019 | N = 249 HIV-positive mothers | Multivariate logistic regression | Non-EBF vs EBF*** | AOR = 0.03 (0.004-0.24) | P = 0.001 | Negative | Variables with P < 0.2 in bivariable analysis; exact covariates not reported. |
| Hospital delivery  (Health facility vs Home) | Mutawulira et al., 2022 | N = 108  HIV-positive mothers | Multivariate logistic regression | EBF vs MF | AOR = 1.98 (1.30-0.70) | P = 0.04 | Positive | Variables with P < 0.05 in bivariable analysis; exact covariates not reported. |
| Hospital delivery  (Health facility vs Home) | Mekonnen et al., 2022 | N = 355 HIV-positive mothers | Multivariate logistic regression | EBF/ERF vs MF | AOR = 0.02 (0.002-0.12) | P < 0.05 | Negative | Variables with P < 0.20 in bivariable analysis; exact covariates not reported. |
| Higher income |  |  |  |  |  |  |  |  |
| Higher income per day  (>= 1 USD vs < 1 USD) | Faustine & Moshi, 2022 | N = 372 HIV-positive mothers | Multivariate logistic regression | EBF vs non-EBF | AOR = 1.83  (1.14-2.94) | P = 0.013 | Positive | Variables with P < 0.05 in bivariable analysis; exact covariates not reported. |
| Higher income per month  (> 59 USD vs <= 29 USD) | Mutawulira et al., 2022 | N = 108  HIV-positive mothers | Multivariate logistic regression | EBF vs MF | AOR = 1.30 (1.15-10.6) | P = 0.013 | Positive | Variables with P < 0.05 in bivariable analysis; exact covariates not reported. |
| High socioeconomic status  (Top vs Lowest) | Napyo et al., 2020 | N = 466 mother-infant pairs | Multivariate Poisson regression | Non-EBF vs EBF*** | ARR = 1.5 (1.01-2.1) | P < 0.05 | Negative | Variables with P < 0.25 in bivariable analysis; exact covariates not reported. |
| Postnatal care |  |  |  |  |  |  |  |  |
| Postnatal follow-up  (Yes vs No) | Dagnew et al., 2019 | N = 213 | Multivariate logistic regression | EBF/ERF vs MF | AOR = 6.9 (4.2-9.3) | P < 0.05 | Positive | Variables with P < 0.05 in bivariable analysis; exact covariates not reported. |
| Postnatal care follow up (No vs Yes)** | Zewdu et al., 2023 | N = 423 HIV-positive mothers | Multivariate logistic regression | MF vs EBF*** | AOR = 1.81 (1.13-2.92) | P = 0.014 | Positive | Variables with p < 0.25 in bivariable analysis; exact covariates not reported. |
| >= 1 Postnatal care  (Yes vs No) | Ejara et al., 2018 | N = 283  HIV-positive mothers | Multivariate logistic regression | Non-EBF vs EBF*** | AOR = 0.18 (0.04-0.81) | P < 0.05 | Positive | Attitude on EBF, mother’s breast problem, infant had mouth ulcers, disclosure of HIV status, maternal education, place of delivery, infant feeding counselling, antenatal care. |
| Higher maternal education |  |  |  |  |  |  |  |  |
| Higher maternal education  (Secondary and above vs Unable to read and write) | Dagnew et al., 2019 | N = 213 | Multivariate logistic regression | EBF/ERF vs MF | AOR = 3.6 (4.2-9.5) | P < 0.05 | Positive | Variables with P < 0.05 in bivariable analysis; exact covariates not reported. |
| Higher maternal education  (Completed secondary education vs No education) | Muhammed & Seid, 2019 | N = 249 HIV-positive mothers | Multivariate logistic regression | Non-EBFvs EBF*** | AOR = 10.35 (1.30-82.63) | P = 0.03 | Negative | Variables with P < 0.2 in bivariable analysis; exact covariates not reported. |
| Higher maternal education  (Grade 12 and less vs higher education)** | Goon et al., 2021 | N = 469 HIV-positive mothers | Multivariate logistic regression | EBF vs non-EBF | AOR = 2.76 (1.02-7.49) | P < 0.05 | Negative | Variables with P < 0.05 in bivariable analysis; exact covariates not reported. |
| ART adherence |  |  |  |  |  |  |  |  |
| ART adherence (No vs Yes)** | Napyo et al., 2020 | N = 466 mother-infant pairs | Multivariate Poisson regression | Non-EBFvs EBF*** | ARR = 1.3 (1.01-1.7) | P < 0.05 | Positive | Variables with P < 0.25 in bivariable analysis; exact covariates not reported. |
| ART adherence (No vs Yes)** | Zewdu et al., 2023 | N = 423 HIV-positive mothers | Multivariate logistic regression | MF vs EBF*** | AOR = 1.99 (1.13-3.50) | P = 0.017 | Positive | Variables with p < 0.25 in bivariable analysis; exact covariates not reported. |
| Breast problems |  |  |  |  |  |  |  |  |
| Experienced breast problems  (No vs Yes)** | Faustine & Moshi, 2022 | N = 372 HIV-positive mothers | Multivariate logistic regression | EBF vs non-EBF | AOR = 3.91  (1.89-8.08) | P < 0.001 | Negative | Variables with P < 0.05 in bivariable analysis; exact covariates not reported. |
| Experienced breast problems  (Yes vs No) | Ejara et al., 2018 | N = 283  HIV-positive mothers | Multivariate logistic regression | Non-EBF vs EBF*** | AOR = 4.89 (1.54-15.60) | P < 0.05 | Negative | Attitude on EBF, infant had mouth ulcers, disclosure of HIV status, maternal education, place of delivery, postnatal care, infant feeding counselling, antenatal care. |
| Employment |  |  |  |  |  |  |  |  |
| Occupation (Employed vs Unemployed) | Muhammed & Seid, 2019 | N = 249 HIV-positive mothers | Multivariate logistic regression | Non-EBF vs EBF | AOR = 4.36 (2.32-8.19) | P = 0.001 | Negative | Variables with P < 0.2 in bivariable analysis; exact covariates not reported. |
| Occupation in the past 12 months  (Unemployed vs employed)** | Goon et al., 2021 | N = 469 HIV-positive mothers | Multivariate logistic regression | EBF vs non-EBF | AOR = 1.66 (1.08-2.56) | P < 0.05 | Negative | Variables with P < 0.05 in bivariable analysis; exact covariates not reported. |
| Pregnancy complications |  |  |  |  |  |  |  |  |
| Problem during pregnancy  (No vs Yes)** | Mekonnen et al., 2022 | N = 355 HIV-positive mothers | Multivariate logistic regression | EBF/ERF vs MF | AOR = 2.61 (1.23-7.96) (Pregnancy complications as reference group) | P < 0.05 | Negative | Variables with P < 0.20 in bivariable analysis; exact covariates not reported. |
| History of obstetric problem  (No vs Yes)** | Genetu et al., 2016 | N = 367  Mother-child pairs | Multivariate logistic regression | EBF vs non-EBF | AOR = 3.4 (1.3-8.9) | P < 0.05 | Negative | Variables with P < 0.20 in bivariable analysis; exact covariates not reported. |
| Social support |  |  |  |  |  |  |  |  |
| Social support (Yes vs No) | Mekonnen et al., 2022 | N = 355 HIV-positive mothers | Multivariate logistic regression | EBF/ERF vs MF | AOR = 1.82 (1.43-6.55) | P < 0.05 | Positive | Variables with P < 0.20 in bivariable analysis; exact covariates not reported. |
| Functional Social Support  (score on scale) | Etowa et al., 2021 | N = 690  HIV-positive women | Multinomial logistic regression | EBF vs MF | RRR = 1.1 | P < 0.05 | Positive | National policy guideline, maternal age, marital status, number of children born after HIV diagnosis, maternal education, maternal employment, baby's father attitude, family’s attitude, cultural beliefs, antenatal care, source of prenatal care, perceived stress, infant feeding attitude |
| More than one child |  |  |  |  |  |  |  |  |
| More pregnancies while HIV positive  (>= 2 vs 1) | Philemon et al., 2022 | N = 524 mother-infant pairs | Multivariate logistic regression | EBF vs non-EBF | AOR = 1.97 (1.10-3.53) | P = 0.02 | Positive | Exact covariates not reported. |
| Delivery supervised by the health worker |  |  |  |  |  |  |  |  |
| Delivery supervised by the health worker  (No vs Yes)** | Napyo et al., 2020 | N = 466 mother-infant pairs | Multivariate Poisson regression | Non-EBFvs EBF*** | ARR = 1.6 (1.01-2.7) | P < 0.05 | Positive | Variables with P < 0.25 in bivariable analysis; exact covariates not reported. |
| HIV positive for less than 5 years |  |  |  |  |  |  |  |  |
| Shorter duration of HIV positive  (> 5 years vs <= 5 years)** | Philemon et al., 2022 | N = 524 mother-infant pairs | Multivariate logistic regression | EBF vs non-EBF | AOR = 0.41 (0.23-0.74) | P = 0.003 | Positive | Exact covariates not reported. |
| Older maternal age (years) |  |  |  |  |  |  |  |  |
| Older maternal age (years)  (18-24 vs >= 35)**  (25-29 vs >= 35)**  (30-34 vs >= 35)** | Mekonnen et al., 2022 | N = 355 HIV-positive mothers | Multivariate logistic regression | EBF/ERF vs MF | AOR = 2.34 (1.26-10.56)  AOR = 3.33 (1.45-8.75)  AOR = 1.51 (1.02-9.85) | P < 0.05  P < 0.05  P < 0.05 | Negative | Variables with P < 0.20 in bivariable analysis; exact covariates not reported. |
| Urban residence |  |  |  |  |  |  |  |  |
| Urban residence  (Rural vs Urban)** | Mekonnen et al., 2022 | N = 355 HIV-positive mothers | Multivariate logistic regression | EBF/ERF vs MF | AOR = 8.08 (2.45-21.62)  (Urban residence as reference group) | P < 0.05 | Negative | Variables with P < 0.20 in bivariable analysis; exact covariates not reported. |
| Alcohol consumption |  |  |  |  |  |  |  |  |
| Alcohol consumption in the past year (Yes vs No) | Goon et al., 2021 | N = 469 HIV-positive mothers | Multivariate logistic regression | EBF vs non-EBF | AOR = 0.54 (0.34-0.85) | P < 0.05 | Negative | Maternal age, marital status, smoking, adherence to antiretroviral therapy, duration of HIV, partner’s HIV disclosure status, maternal education, mother’s employment. |
| Frequent early infant diagnosis clinic attendance |  |  |  |  |  |  |  |  |
| Frequent early infant diagnosis clinic attendance  (4-6 times vs 1-3 times)  (> 6 times vs 1-3 times) | Mutawulira et al., 2022 | N = 108  HIV-positive mothers | Multivariate logistic regression | EBF vs MF | AOR = 0.30  (0.09-1.0)    AOR = 0.12 (0.02-0.70) | P = 0.05    P= 0.019 | Negative | Variables with P < 0.05 in bivariable analysis; exact covariates not reported. |
| Increased child age |  |  |  |  |  |  |  |  |
| Increased child age  (7-12 months vs 0-6 months) | Mutawulira et al., 2022 | N = 108  HIV-positive mothers | Multivariate logistic regression | EBF vs MF | AOR = 0.71 (0.21-0.99) | P = 0.04 | Negative | Variables with P < 0.05 in bivariable analysis; exact covariates not reported. |
| Infant had mouth ulcers |  |  |  |  |  |  |  |  |
| Infant had mouth ulcers (Yes vs No) | Ejara et al., 2018 | N = 283  HIV-positive mothers | Multivariate logistic regression | Non-EBF vs EBF*** | AOR = 6.41 (2.07-19.85) | P < 0.05 | Negative | Attitude on EBF, mother’s breast problem, disclosure of HIV status, maternal education, place of delivery, postnatal care, infant feeding counselling, antenatal care. |
| Stress |  |  |  |  |  |  |  |  |
| Perceived Stress (score on Cohen’s scale) | Etowa et al., 2021 | N = 690  HIV-positive women | Multinomial logistic regression | EBF vs MF | RRR = 0.9 | P < 0.05 | Negative | National policy guideline, maternal age, marital status, number of children born after HIV diagnosis, maternal education, maternal employment, baby's father attitude, family’s attitude, cultural beliefs, antenatal care, source of prenatal care, functional social support, infant feeding attitude |
| *For studies that reported statistically significant findings but did not provide exact p-values, the significance level was recorded as p < 0.05.  **Studies used the predictor group as the reference group.  ***Studies treated non-EBF or MF as the outcome.  Abbreviations: CI = confidence interval; EBF = exclusive breastfeeding; ERF = exclusive replacement feeding; AOR = adjusted odds ratio; MF = mixed feeding; RRR = relative risk ratio; IFP = infant feeding practice; ARR = adjusted risk ratio. | | | | | | | | |
